# Incidence and treatment-modifying impact of immune checkpoint inhibitor–associated peripheral edema: a systematic review and meta-analysis

**DOI:** 10.1093/jncics/pkag058

**Published:** 2026-06-03

**Authors:** Anneliese Markus, Kazuya Tsuchiya, Toshiaki Takahashi, Evelyn Elias MBBCh, Mako Koseki, Yoshito Nishimura, Nicholas C Rohs, Yu Fujiwara

**Affiliations:** Department of Medicine, Roswell Park Comprehensive Cancer Center, Buffalo, NY, United States; Department of Medicine, Jacobs School of Medicine and Biomedical Sciences, Buffalo, NY, United States; Department of Internal Medicine, Morehouse School of Medicine, Atlanta, GA, United States; Department of Internal Medicine, John A. Burns School of Medicine, University of Hawaii, Honolulu, HI, United States; Department of Medicine, Icahn School of Medicine at Mount Sinai, Mount Sinai Morningside/West, New York, NY, United States; Department of Medicine, Icahn School of Medicine at Mount Sinai, Mount Sinai Morningside/West, New York, NY, United States; Division of Hematology and Oncology, Mayo Clinic, Rochester, MN, United States; Center for Thoracic Oncology, Tisch Cancer Institute, Icahn School of Medicine at Mount Sinai, New York, NY, United States; Department of Medicine, Roswell Park Comprehensive Cancer Center, Buffalo, NY, United States; Department of Medicine, Jacobs School of Medicine and Biomedical Sciences, Buffalo, NY, United States; Department of Clinical Oncology, Institute of Science Tokyo, Tokyo, Japan

## Abstract

**Background:**

Immune-related adverse events disrupt immune checkpoint inhibitor therapy and impact quality of life. Peripheral edema is common among patients with cancer, however the causality of peripheral edema can be unclear in the context of multidrug regimens.

**Methods:**

PubMed/MEDLINE, Embase, and Web of Science were searched to identify phase 3 randomized controlled trials (RCTs) evaluating immune checkpoint inhibitors reporting peripheral edema incidence. The incidence of edema was calculated according to adverse event, immune checkpoint inhibitor, and treatment patterns. A random-effects meta-analysis pooled odds ratios (ORs) of edema incidence between immune checkpoint inhibitor and non–immune checkpoint inhibitor groups. A systematic review characterizing the interventions for immune-related peripheral edema was also conducted.

**Results:**

From 58 RCTs (22 590 patients), treatment-related peripheral edema (grade 1-5) occurred in 2.80% (*n* = 195 of 6969) of patients receiving immune checkpoint inhibitor monotherapy, 4.96% (*n* = 112 of 2257) of patients receiving immune checkpoint inhibitor with chemotherapy, and 8.73% (n = 205 of 2349) of patients receiving immune checkpoint inhibitor with targeted therapy. Meta-analysis showed immune checkpoint inhibitor monotherapy had lower treatment-related peripheral edema incidence compared with chemotherapy (OR = 0.45, 95% CI = 0.27 to 0.73; *P *= .001, *I*^2^ = 69%) with the lowest relative incidence among programmed cell death protein 1 vs chemotherapy (OR = 0.41, 95% CI = 0.23 to 0.73; *P *= .003) and immune checkpoint inhibitor vs taxane chemotherapy (OR = 0.32, 95% CI = 0.18 to 0.58; *P *< .001). A systematic review of treatment outcomes for immune-related peripheral edema among 40 patients (19 studies) revealed 82.5% (*n* = 33 of 40) of cases received glucocorticoid treatment, and immune checkpoint inhibitors were permanently discontinued in 35% (*n* = 14 of 40).

**Conclusions:**

Peripheral edema is a rare but potentially serious adverse event associated with immune checkpoint inhibitors. Immune checkpoint inhibitors likely confer less peripheral edema risk than taxane therapy. To avoid unnecessary treatment interruptions, treatment discontinuation requires careful consideration, particularly when edema develops following immune checkpoint inhibitor combination therapy.

## Introduction

Over the past few decades, immune checkpoint inhibitors have revolutionized cancer treatment and demonstrated a clinically significant long-term survival benefit across multiple tumor types. Immune checkpoint inhibitors block cell surface proteins such as programmed death protein-1 (PD-1), programmed death-ligand-1 (PD-L1), and cytotoxic T-lymphocyte antigen-4 (CTLA-4), which normally function as co-inhibitory immune checkpoints to prevent overactivation of the immune system.^1^^,^^2^ Cancer cells can exploit these pathways to suppress immune responses and avoid detection.^2^ Accordingly, blockade of co-inhibitory immune checkpoints restores the immune system’s ability to recognize and attack tumor cells, leading to significant improvements in survival outcomes across various malignancies, including melanoma, non-small cell lung cancer, and renal cell carcinoma.^1^^,^^2^

Activation of the immune system by immune checkpoint inhibitors can, however, lead to unintended nontumor-specific immunoreactivity, known as immune-related adverse events. Immune-related adverse events can affect nearly any organ system with the most common manifestations involving the skin, gastrointestinal tract, liver, and endocrine glands.^3^^,^^4^ Because immune-related adverse events can range from mild to life-threatening, it is critical to delineate the safety and toxicity of immune checkpoint inhibitors for cancer treatment. In the context of multidrug treatment regimens, determining the causality of adverse events can be challenging and may lead physicians to mistakenly disrupt or discontinue a medication unnecessarily.^5-8^

One adverse event of particular interest is the severe generalized edema occasionally associated with immune checkpoint inhibitors. Although immune-related peripheral edema is uncommon and almost never life-threatening, its occurrence poses important clinical challenges, including diagnostic uncertainty, symptomatic burden, and treatment discontinuation.^7-9^ Significant peripheral edema in the setting of immune checkpoint inhibitors is posited to be secondary to increased vascular permeability and endothelial damage by inflammatory and dysregulated immune conditions following immune checkpoint inhibitor treatments. Primary immune checkpoint inhibitor edema is a diagnosis of exclusion, characterized by weight gain and physical exam findings with or without hypoalbuminemia in the absence of known cardiac or liver failure, renal protein leak or renal failure, exudative enteropathy, or monoclonal gammopathy. Presentations resembling capillary leakage syndrome possibly secondary to CD8-mediated endothelial cell damage and veno-occlusive disease or sinusoidal obstruction syndrome have been reported.^7^^,^^8^ Crucially, the incidence and management of this phenomenon are complicated by the large percentage of patients receiving immune checkpoint inhibitors alongside another agent.

Thus, this study is a systematic review and meta-analysis aiming to investigate the frequency and severity of peripheral edema occurrence across immune checkpoint inhibitor monotherapy and various immune checkpoint inhibitor combination therapies. Additionally, we aimed to summarize current management strategies and clinical outcomes of immune-related peripheral edema in patients with solid tumors receiving immune checkpoint inhibitors, with a particular focus on rates of immune checkpoint inhibitor discontinuation.

## Methods

### Data sources and searches

The Preferred Reporting Items for Systematic Reviews and Meta-analyses 2020 statement guidelines were followed in conducting this systematic review and meta-analysis. Our research protocol was prospectively registered in PROSPERO (ID CRD42022379374).

Articles were retrieved from PubMed/MEDLINE, Embase, and Web of Science to achieve 2 aims: (1) identify the incidence and relative incidence rates of immune checkpoint inhibitor–associated peripheral edema and (2) summarize management and treatment outcomes for immune-related peripheral edema. The search strategy for edema incidence is detailed in Table S1, and for immune-related edema treatment is detailed in Table S2. The last date for the literature search is February 27, 2023.

### Definition of edema as an adverse event

Adverse events reported in randomized controlled trials (RCTs) were reported and graded 1-5 according to the definition of the Common Terminology Criteria for Adverse Events (CTCAE) published by the National Cancer Institute. The CTCAE version was according to each RCT’s individual usage (Methods S1).

Adverse events were classified into treatment-related adverse event, immune-related adverse event, and any-cause by each study’s investigators. Treatment-related adverse events are adverse events judged by the study’s investigators as likely attributed to the investigational agent(s) a patient received. In contrast, immune-related adverse events are adverse events judged by the study’s investigators as attributed to the patient’s immune system regardless of treatment attribution. Finally, any-cause adverse events are any adverse event occurring during the study period, regardless of treatment attribution. Given the differing adverse event definitions, incidence rate calculations and meta-analyses of treatment-related adverse event, immune-related adverse event, and any-cause peripheral edema were performed separately.

### Selection criteria

To identify the incidence rates of immune checkpoint inhibitor–associated peripheral edema, we selected phase 3 RCTs evaluating immune checkpoint inhibitors in patients with solid tumors that reported the incidence of peripheral edema (treatment-related adverse event, immune-related adverse event, any-cause). To summarize the management and outcomes of immune-related peripheral edema, we searched for case reports, series, cohorts, and clinical trials that reported immune checkpoint inhibitor–related edema treatment. Detailed selection criteria can be found in Table S3.

Meta-analysis of relative peripheral edema incidence consisted of only RCTs with the design “ICI vs A” or “ICI + A vs A,” where the experimental group was immune checkpoint inhibitor (ICI) monotherapy or combination therapy, and “A” represents the treatment administered to the control group (chemotherapy, placebo, etc.). Randomized controlled trials with the design “ICI vs A” were selected to compare the effects of immune checkpoint inhibitor therapy with systemic therapy on peripheral edema incidence. Similarly, RCTs with a study design “ICI + A vs A” were selected to model the effect of the addition of one immune checkpoint inhibitor to systemic therapy on peripheral edema incidence.

### Data extraction and risk of bias assessment

Two independent investigators extracted data from eligible studies, including study characteristics, the number of patients in each arm, and the incidence and type of adverse events. Disagreements were resolved by consensus with a third investigator. Cochrane Risk of Bias Assessment Tool V2 was used^10^ (Figure S1). Details of data extraction and risk of bias assessment are given in Supplementary Methods S1.

### Data synthesis and statistical analysis

Incidence of treatment-related adverse event, any-cause adverse event, and immune-related adverse event peripheral edema was calculated. Among each adverse event grouping of peripheral edema (treatment-related, immune-related, any-cause), incidence was categorized by edema grade (any grade or grade 3-5), treatment class, and immune checkpoint inhibitor type. A meta-analysis using random effects modeling to pool odds ratios (ORs) was employed to evaluate the contribution of immune checkpoint inhibitor therapy to peripheral edema incidence, relative to and in addition to other systemic therapies. Exploratory random-effects meta-regression analyses were performed to examine whether grade 1-5 any-cause edema incidence or grade 1-5 treatment-related edema incidence was related to treatment discontinuation or interruption rates across treatment arms. Univariable models included edema incidence as the sole moderator, whereas multivariable models additionally included median follow-up duration as a covariate. These analyses were restricted to treatment arms with available follow-up data. Significance was set for equivalence hypothesis testing using the 2-tailed 0.05 level. *I*^2^ statistics were used to assess heterogeneity, and *I*^2^ greater than 50% was considered statistically significant heterogeneity. We used Review Manager (Revman) version 5.4 (Cochrane Collaboration, London, United Kingdom) for statistical analysis and figure generation. Plotly version 5.24.1 (Plotly Technologies Inc. Collaborative data science. Montréal, QC, 2015) was additionally used for figure generation.

## Results

### Systematic review and meta-analysis of immune checkpoint inhibitor–related peripheral edema incidence

#### Literature search results

**Figure 1.**
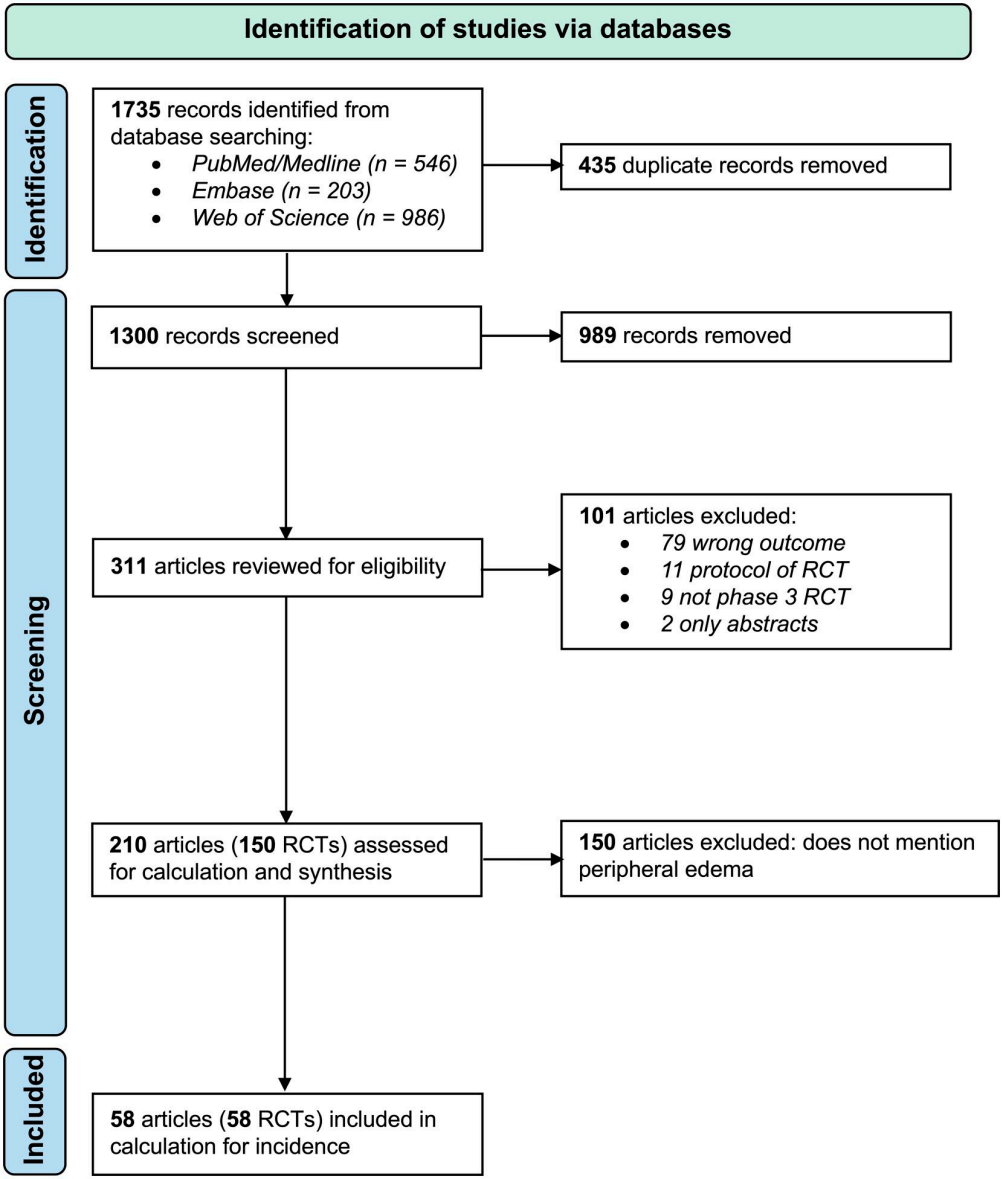
Flowchart of the systematic review process for incidence of peripheral edema. Based on the initial screening and eligibility assessment, 58 studies were identified. Abbreviation: RCT = randomized controlled trial.

Our literature search identified 1735 potential articles from which 435 articles were removed because of duplicates, and 1300 studies underwent further screening. Of these, 58 phase 3 RCTs met the inclusion criteria and were not excluded based on the criteria listed in Table S3. These studies comprised a cohort of 22 590 patients with solid tumors who received immune checkpoint inhibitor monotherapy or combination therapy. A flowchart of the study selection process is shown in Figure 1. All included RCTs can be found in References S1.

#### Characteristics of identified studies

Among the 58 selected phase 3 RCTs comprising 22 590 solid tumor patients who received immune checkpoint inhibitor monotherapy or combination therapy, classification of peripheral edema as an adverse event was distributed between treatment-related adverse events, immune-related adverse events, and any-cause adverse events. Specifically, 33 studies comprising 12 962 (57.38%) patients reported treatment-related peripheral edema outcomes, 23 studies comprising 9123 (40.38%) patients reported any-cause peripheral edema, and 2 studies comprising 505 (2.20%) patients reported immune-related peripheral edema outcomes.

The studies included 24 RCTs of immune checkpoint inhibitor monotherapy composed of 10 323 (45.70%) patients, 18 RCTs of immune checkpoint inhibitor therapy combined with chemotherapy composed of 6775 (30.00%) patients, and 9 RCTs of immune checkpoint inhibitor therapy combined with molecular targeted therapy composed of 3335 (14.76%) patients. A limited number of RCTs studied dual immune checkpoint inhibitor therapy (*n* = 1113 [4.93%]), dual immune checkpoint inhibitor therapy with chemotherapy (*n* = 626 [2.77%]), or immune checkpoint inhibitor with vaccine (*n* = 345 [1.53%]).

Within each treatment classification, immune checkpoint inhibitor type was spread between PD-1, PD-L1, and CTLA-4. A total of 11 279 (49.93%) patients received PD-1 inhibitors, 8538 (37.8%) patients received PD-L1 inhibitors, and 1034 (4.58%) patients received CTLA-4 inhibitors. Combinations such as PD-1 with CTLA-4 or PD-L1 with CTLA-4 represented 927 (4.10%) and 812 (3.59%) patients, respectively.

**Figure 2.**
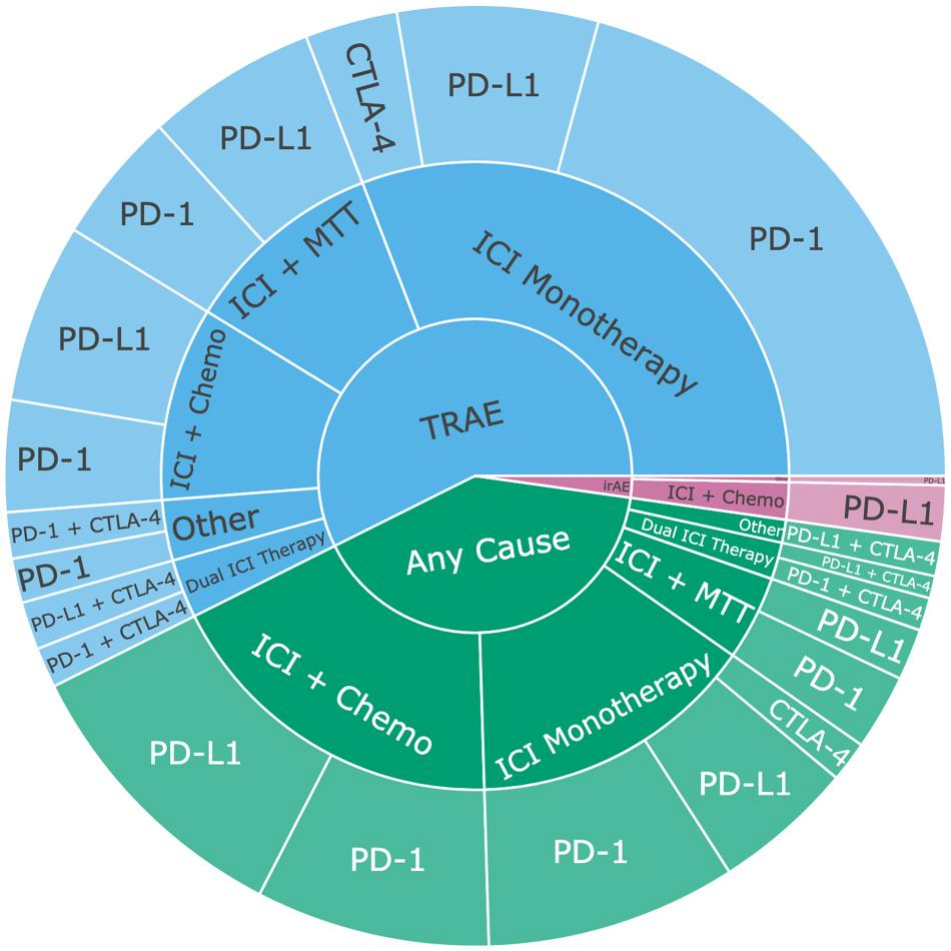
Sunburst plot of distribution of study patients between adverse event, treatment, and ICI type classifications. Other represents dual ICI + chemo, ICI + MTT + chemo, and ICI + vaccine. Abbreviations: Chemo = chemotherapy; CTLA-4 = cytotoxic T-lymphocyte antigen-4; ICI = immune checkpoint inhibitor; irAE = immune-related adverse event; MTT = molecular-targeted therapy; PD-1 = programmed death protein-1; PD-L1 = programmed death-ligand-1; TRAE = treatment-related adverse event.

A detailed table of individual study characteristics is found in Table S4. Figure 2 demonstrates the distribution of study patients between adverse event, treatment, and immune checkpoint inhibitor type classifications. The summary of the risk of bias for each trial is shown (Figure S1). Among the 58 RCTs, 33 were open-label, 24 were double-blind, and 1 was triple-blind. Although the open-label trials raised some concerns regarding potential bias in outcome measurement, the overall risk of bias across all studies was relatively low.

#### Immune checkpoint inhibitor–related peripheral edema incidence

The incidence of treatment-related adverse event, immune-related adverse event, and any-cause peripheral edema was analyzed separately. Among each adverse event grouping of peripheral edema (treatment-related, immune-related, any-cause), incidence is categorized by treatment class and immune checkpoint inhibitor type.

The total incidence of treatment-related grade 1-5 peripheral edema was 4.01% (*n* = 520 of 12 962). Among patients receiving immune checkpoint inhibitor monotherapy, the incidence of peripheral edema of PD-1, PD-L1, and CTLA-4 inhibitors was 2.21% (*n* = 104 of 4694), 3.72% (*n* = 58 of 1557), and 4.60% (*n* = 33 of 718), respectively. Immune checkpoint inhibitor therapy combined with chemotherapy or with molecular-targeted therapy exhibited overall higher incidence of treatment-related peripheral edema, with overall rates of 4.96% (*n* = 112 of 2257) and 8.73% (*n* = 205 of 2349), respectively. Few events were observed in dual immune checkpoint inhibitor therapy (*n* = 0 of 684) or dual immune checkpoint inhibitor with chemotherapy (0.0056%; *n* = 2 of 358) (Table 1).

**Table 1.** Incidence of treatment-related and any-cause peripheral edema.^a^

Treatment Class	ICI Type	Treatment-related peripheral edema				Any-cause peripheral edema			
		No.	RCTs	Grade 1-5 No. (%)	Grade 3-5 No. (%)	No.	RCTs	Grade 1-5 No. (%)	Grade 3-5 No. (%)
ICI monotherapy	Subtotal	6969	21	195 (2.8)	13 (0.2)	3354	10	213 (6.4)	3 (0.1)
	PD-1	4694	15	104 (2.2)	6 (0.1)	1946	6	124 (6.4)	3 (0.2)
	PD-L1	1557	4	58 (3.7)	1 (0.1)	1092	4	88 (8.1)	0 (0)
	CTLA-4	718	3	33 (4.6)	6 (0.8)	316	2	1 (0.3)	0 (0)
Dual ICI therapy	Subtotal	684	2	0 (0)	0 (0)	429	2	34 (7.9)	3 (0.7)
	PD-1 + CTLA-4	313	1	0 (0)	0 (0)	256	1	27 (10.5)	2 (0.8)
	PD-L1 + CTLA-4	371	1	0 (0)	0 (0)	173	1	7 (4.0)	1 (0.6)
ICI + chemotherapy	Subtotal	2257	7	112 (5.0)	4 (0.2)	4086	10	550 (13.4)	10 (0.2)
	PD-1	866	3	77 (8.9)	2 (0.2)	1816	4	266 (14.6)	3 (0.2)
	PD-L1	1391	4	35 (2.5)	2 (0.1)	2270	6	284 (12.5)	7 (0.3)
ICI + molecular-targeted therapy	Subtotal	2349	7	205 (8.7)	4 (0.2)	986	4	142 (14.4)	4 (0.4)
	PD-1	1025	3	52 (5.1)	2 (0.2)	587	2	72 (12.3)	1 (0.2)
	PD-L1	1324	4	153 (11.6)	2 (0.2)	399	2	70 (17.5)	3 (0.8)

Abbreviations: CTLA-4 = cytotoxic T-lymphocyte antigen-4; ICI = immune checkpoint inhibitor; PD-1 = programmed death protein-1; PD-L1 = programmed death-ligand-1; RCT = randomized controlled trial.

a Dual ICI + chemo, ICI + MTT + chemo, and ICI + vaccine excluded; see Tables S4 and S5.

The total incidence of any-cause grade 1-5 peripheral edema was 10.29% (*n* = 940 of 9123). Among patients receiving immune checkpoint inhibitor monotherapy, the incidence of peripheral edema in PD-1, PD-L1, and CTLA-4 inhibitor use was 6.37% (*n* = 124 of 1946), 8.06% (*n* = 88 of 1092), and 0.32% (*n* = 1 of 316), respectively. Similar to treatment-related peripheral edema, incidence rates of any-cause peripheral edema were greater in patients receiving combinations of immune checkpoint inhibitor therapy plus chemotherapy or molecular-targeted therapy, with rates of 13.46% (*n* = 550 of 4086) and 14.40% (*n* = 142 of 986), respectively. Fewer events were observed in dual immune checkpoint inhibitor therapy (7.93%; *n* = 34 of 429) or dual immune checkpoint inhibitor and chemotherapy (0.37%; *n* = 1 of 268) (Table 1).

Nonimmune checkpoint inhibitor-containing regimens demonstrated a grade 1-5 treatment-related peripheral edema incidence of 6.2% (*n* = 340 of 5482) and an any-cause incidence of 12.5% (*n* = 421 of 3375). Immune checkpoint inhibitor-containing regimens demonstrated an overall grade 1-5 treatment-related peripheral edema incidence of 4.0% (*n* = 1034 of 25 579) and an any-cause incidence of 10.3% (*n* = 1878 of 18 246). Incidence rates observed in immune checkpoint inhibitor–containing combination regimens likely represent a mixture of primary immune-related edema and secondary edema attributable to nonimmune checkpoint inhibitor agents (Table S5).

**Figure 3.**
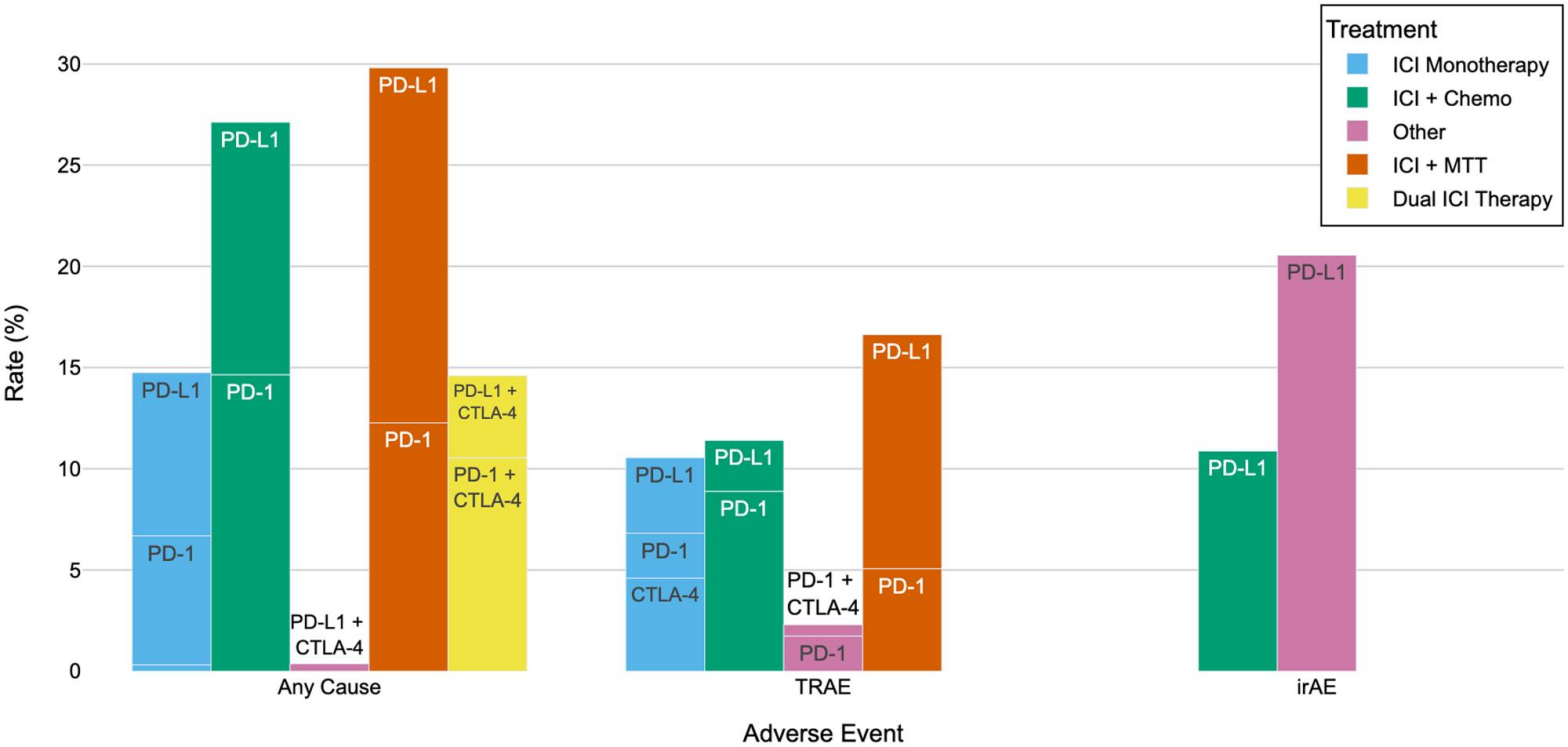
Stacked barplot of grade 1-5 peripheral edema incidence rate by adverse event classification, ICI type, and treatment category. Other represents dual ICI + chemo, ICI + MTT + chemo, and ICI + vaccine. Abbreviations: chemo = chemotherapy; CTLA-4 = cytotoxic T-lymphocyte antigen-4; ICI = immune checkpoint inhibitor; irAE = immune-related adverse event; MTT = molecular-targeted therapy; PD-1 = programmed death protein-1; PD-L1 = programmed death-ligand-1; TRAE = treatment-related adverse event.

The overall incidence of grade 3-5 peripheral edema was 0.18% (*n* = 23 of 12 962) and 0.23% (*n* = 21 of 9123) for treatment-related and any-cause peripheral edema, respectively (Table 1). The overall rate of grade 1-5 immune-related peripheral edema was 12.27% (*n* = 62 of 505), with no grade 3-5 cases reported (*n* = 0 of 505). Figure 3 provides a visual representation of the relative incidence of grade 1-5 peripheral edema across major treatment classes by adverse event classification and immune checkpoint inhibitor type. Complete incidence data across all treatment and adverse event schemas is reported in Tables S5 and S6.

#### Meta-analysis of immune checkpoint inhibitor–related peripheral edema incidence

**Figure 4.**
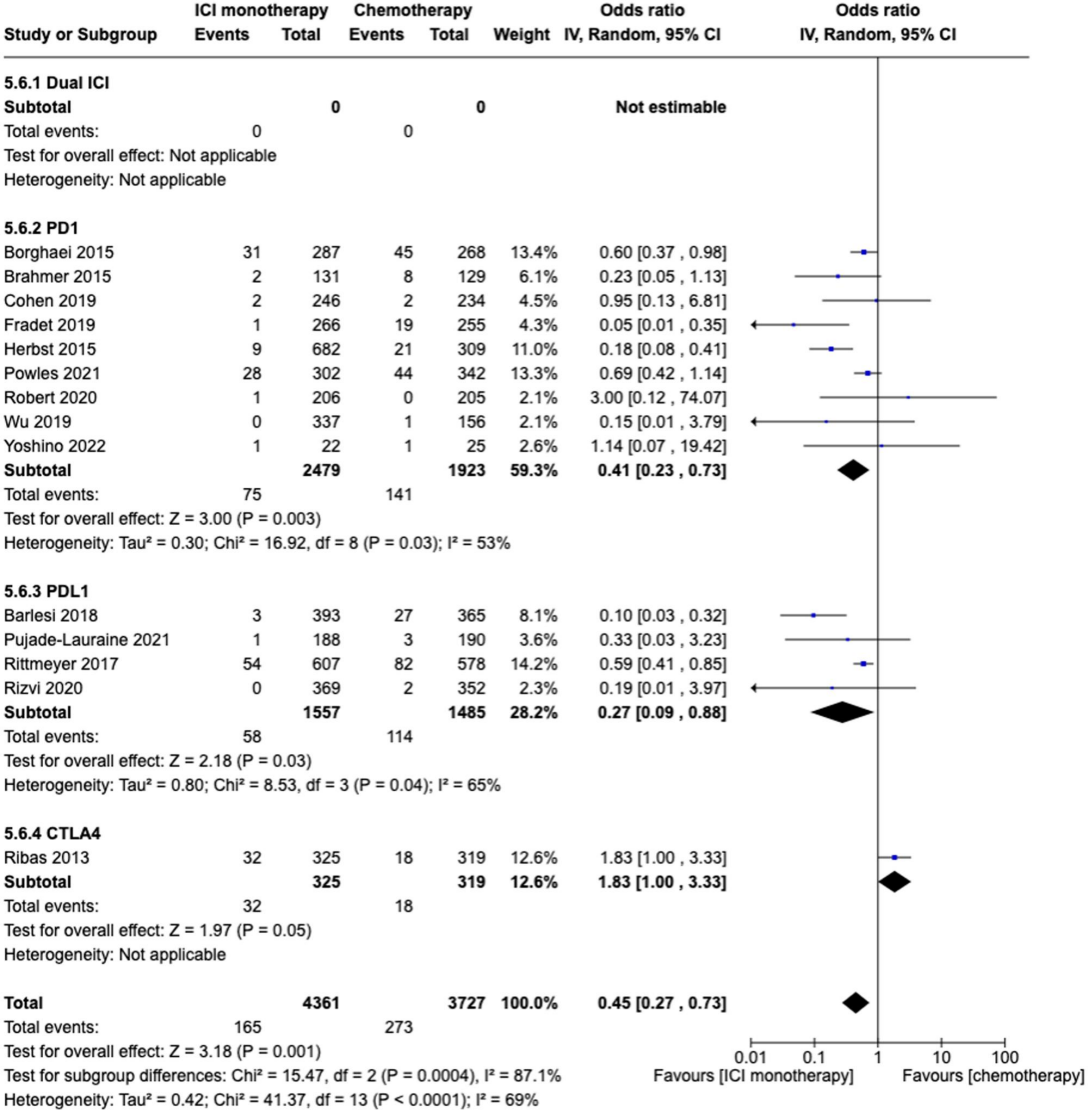
Pooled odds ratio for treatment-related peripheral edema grade 1-5 in patients treated with ICI monotherapy or chemotherapy–subgroup analysis by ICI type. Abbreviations: ICI = immune checkpoint inhibitor; PD1 = programmed death protein-1; PDL1 = programmed death-ligand-1; CTLA4 = cytotoxic T-lymphocyte antigen-4 ; IV = inverse variance.

**Figure 5.**
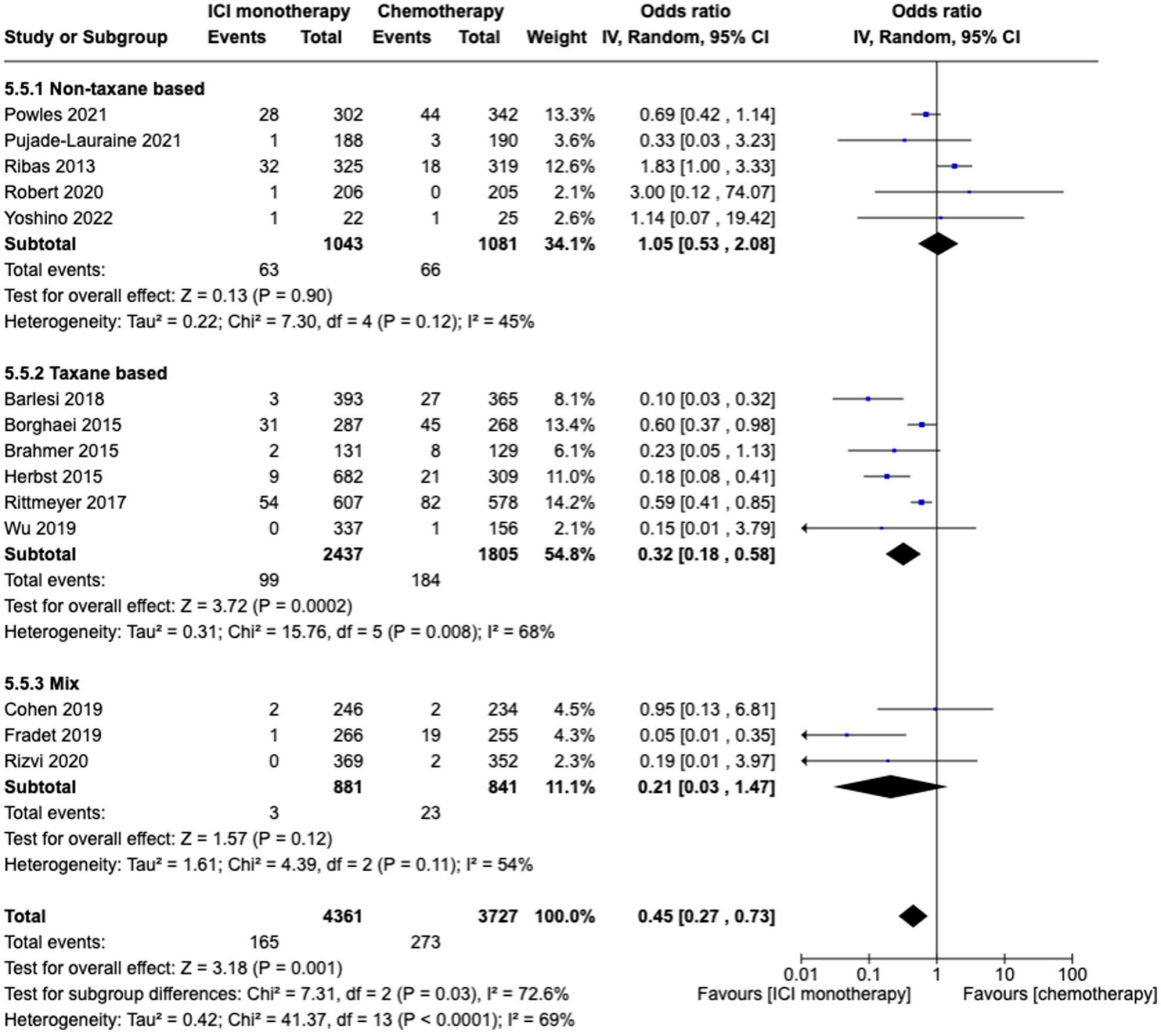
Pooled odds ratio for treatment-related peripheral edema grade 1-5 in patients treated with ICI monotherapy or chemotherapy–subgroup analysis by nontaxane-based and taxane-based (docetaxel, paclitaxel) regimen. Abbreviations: ICI = immune checkpoint inhibitor; PD1 = programmed death protein-1; PDL1 = programmed death-ligand-1; CTLA4 = cytotoxic T-lymphocyte antigen-4; IV = inverse variance.

When compared with chemotherapy, the use of immune checkpoint inhibitor monotherapy was associated with an overall lower incidence of any grade treatment-related peripheral edema (OR = 0.45, 95% CI = 0.27 to 0.45; *P *= .001, *I*^2^ = 69%) (Figure S2). Subgroup analysis of immune checkpoint inhibitor monotherapy vs chemotherapy shows a difference in treatment-related peripheral edema incidence between PD-1, PD-L1, and CTLA-4 immune checkpoint inhibitor types (*P < .*001, *I*^2^ = 87.1%), with the lowest relative incidence among PD-1 vs chemotherapy (OR = 0.41, 95% CI = 0.23 to 0.73; *P *= .003, *I*^2^ = 53%) (Figure 4). Subgroup analysis of immune checkpoint inhibitor monotherapy vs taxane, nontaxane, or mixed chemotherapy reveals a difference between groups (*P < .*001, *I*^2^ = 72.6%), with the lowest relative rates of treatment-related edema in immune checkpoint inhibitor monotherapy vs taxane chemotherapy (OR = 0.32, 95% CI = 0.18 to 0.58; *P < .*001, *I*^2^ = 68%) (Figure 5). Immune checkpoint inhibitor monotherapy had similar rates of treatment-related edema compared with nontaxane chemotherapy (OR = 1.05, 95% CI = 0.53 to 2.08; *P* = .90, *I*^2^ = 45%). It should be noted that subgroup analysis based on immune checkpoint inhibitor subtypes and chemotherapy class both showed high heterogeneity in subgroups. In contrast, no statistically significant difference was found between rates of any-cause peripheral edema in patients treated with immune checkpoint inhibitor monotherapy vs chemotherapy (OR = 0.75, 95% CI = 0.42 to 1.32; *P* = .31, *I*^2^ = 32%) (Figure S3).

The overall add-on effect of immune checkpoint inhibitors to systemic therapy on treatment-related peripheral edema incidence was not statistically significant (OR = 1.15, 95% CI = 0.94 to 1.42; *P* = .18, *I*^2^ = 0%) (Figure S4). Subgroup analyses involving the add-on effect of immune checkpoint inhibitors to chemotherapy (OR = 1.27, 95% CI = 0.93 to 1.72; *P* = .13, *I*^2^ = 0%) (Figure S5) and molecular-targeted therapy (OR = 1.09, 95% CI = 0.82 to 1.45; *P* = .56, *I*^2^ = 47.0%) (Figure S6) were analogous. Similarly, the overall add-on effect of immune checkpoint inhibitors to systemic therapy on any-cause peripheral edema was not statistically significant (OR = 1.17, 95% CI = 0.95 to 1.43; *P* = .14, *I*^2^ = 48%) (Figure S7), including addition to chemotherapy (OR = 1.10, 95% CI = 0.92 to 1.31; *P* = .31, *I*^2^ = 27%) (Figure S8) and to molecular-targeted therapy (OR = 1.56, 95% CI = 0.95 to 2.57; *P* = .08, *I*^2^ = 40) (Figure S9).

### Meta-regression analysis of peripheral edema related treatment discontinuation and interruption

In follow-up adjusted meta-regression, rates of adverse events–related treatment discontinuation was not associated with grade 1-5 any-cause edema incidence at a statistically significant level (β = 0.22, 95% CI = −0.21 to 0.66; *P* = .32), whereas longer follow-up duration was associated with higher adverse events–related discontinuation at a statistically significant level (β = 0.19, 95% CI = 0.01 to 0.37; *P = .*03). Rates of treatment-related discontinuation were jointly associated with grade 1-5 treatment-related edema incidence and follow-up duration (*P = .*047, *I*^2^ = 95%), although neither treatment-related edema (β = 0.33, 95% CI = −0.06 to 1.16; *P = .*08) nor follow-up duration (β = 0.16, 95% CI = −0.01 to 0.33; *P = .*07) was individually associated with discontinuation (Table 2). No statistically significant association was observed for treatment interruption in either univariable or follow-up–adjusted models (Table S7).

**Table 2.** Meta-regression analysis of treatment discontinuation by grade 1-5 any cause/TRAE edema and follow up time.

Outcome x Discontinuation	Type of analysis	No. of treatment arms	Edema coeff. (95% CI for β)	Edema *P*	Follow-up coeff. (95% CI for β)	Follow-up *P*	Overall *P*	*R*²	Residual *I*²
Any edema × AE-related	Meta-regression	21	0.25 (−0.22 to 0.73)	.2931	—	—	.2931	0	0.9176
Any edema × AE-related	Adjusted by follow-up	21	0.22 (−0.21 to 0.66)	.316	0.19 (0.01 to 0.37)	.0304	.0512	0.1704	0.9002
TRAE edema × Treatment-related	Meta-regression	31	0.53 (−0.13 to 1.18)	.1149	—	—	.1149	0.0391	0.9585
TRAE edema × Treatment-related	Adjusted by follow-up	31	0.55 (−0.06 to 1.16)	.0808	0.16 (0.01 to 0.33)	.0661	.0471	0.1494	0.9523

Abbreviations: x = as a linear predictor of, B = regression coefficient, AE = adverse event; coeff. = coefficient; TRAE = treatment-related adverse event.

### Systematic review of management and treatment outcomes of immune-related edema

#### Study selection and characteristics

In total, 457 potential articles were found that reported edema, including peripheral edema, capillary leak syndrome, serositis, efusions of the pleura, peritoneum, and/or pericardium, and anasarca as potential immune-related adverse events. Eventually, 19 articles (17 case reports or series, 1 phase 1-2 clinical trial, and 1 pharmacovigilance study) that specifically reported immune-related peripheral edema as an adverse event were selected for use. A flowchart of the study selection process is shown in Figure S10. All included articles can be found in References S2.

In patients who developed immune-related peripheral edema during immune checkpoint inhibitor therapy, the median age was 61 years (IQR = 52-70 years), and 44% of patients were female (*n* = 15 of 34). The most common cancers were malignant melanoma (*n* = 16 of 40 [40.0%]) and non-small cell lung cancer (*n* = 11 [27.5%]). Of the cases, 72.5% (*n* = 29 of 40) occurred in patients who received PD-1 blockade, followed by PD-L1 blockade in 12.5% (*n* = 5) and dual immune checkpoint inhibitors (CLTA-4 + PD-1 in 7.5% [*n* = 3]). Comorbid serositis such as pleural effusion (*n* = 18 of 40) and ascites (*n* = 14 of 40) were common.

#### Treatment outcomes of immune-related edema

We classified immune-related peripheral edema into primary immune-related peripheral edema (*n* = 21 of 40 [52.5%]), edema secondary to other immune-related adverse events (*n* = 17 of 40 [42.5%]), or unclear (*n* = 2 of 40 [5.0%]). Secondary immune-related adverse events included myocarditis and/or pericarditis, eosinophilic fasciitis, and drug rash with eosinophilia and systemic symptoms syndrome. Glucocorticoids were employed to manage immune-related peripheral edema in 33 (82.5%) patients. Prednisone-equivalent doses of more than 1 mg/kg per day was required in 14 of 21 (66.7%) patients with primary immune-related edema vs only 8 of 17 cases with secondary immune-related edema . Immune checkpoint inhibitor discontinuation was confirmed in 32 (80%) patients, among which 4 of 21 (19.5%) primary immune-related edema patients and 10 of 17 (58.8%) secondary immune-related edema patients were confirmed to have permanently discontinued immune checkpoint inhibitors. Treatment outcomes are summarized in Table 3.

**Table 3.** Summary of management of irAE peripheral edema (total n = 40).

Management or Treatment Outcome	Primary irAE	Secondary irAE	Total
	No. (%)	No. (%)	No. (%)
Steroid use, any dosage	17 (81.0)	16 (94.1)	33 (82.5)
High dose steroid use, ≥1 mg/kg prednisone or equivalent	14 (66.7)	8 (47.0)	22 (55.0)
ICI discontinued, permanently or temporarily	21 (100)	11 (64.7)	32 (80.0)
ICI discontinued permanently	4 (19.5)	10 (58.8)	14 (35.0)
Total	21 (100)	17 (100)	40 (100)

Abbreviations: ICI = immune checkpoint inhibitor; irAE = immune-related adverse event.

## Discussion

Peripheral edema has a considerable negative impact on the quality of life of patients with solid tumors, interfering with activities of daily living such as ambulation, washing, and dressing.^11^^,^^12^ Psycho-emotional effects of peripheral edema include anxiety and/or depression and somatic complaints of pain and/or fatigue.^12^^,^^13^ Beyond these functional and psychological consequences, edema (including peripheral edema) has emerged as a newly recognized and occasionally severe immune-related adverse event.^14^^,^^15^

To our knowledge, this is the largest meta-analysis to date examining edema in the setting of immune checkpoint inhibitor therapy and among the few studies to compare edema rates between immune checkpoint inhibitor monotherapy and combination regimens. This analysis also distinctly separates analysis by grouped investigator-defined modality of adverse event (treatment-related adverse event, any-cause, and immune-related adverse event). In the present study, we find the overall incidence rates of grade 1-5 peripheral edema to be 4.01% (*n* = 520 of 12 962; treatment-related ), 10.29% (*n* = 940 of 9123; any-cause), and 12.27% (*n* = 62 of 505; immune-related).

Fundamental research by Velev et al.^14^ in 2023 reported a 0.19% incidence of immune checkpoint inhibitor–related generalized edema as a new category of adverse events with immune checkpoint inhibitors, with 50% grade 4 or higher and 20% mortality (4 of 20 patients). Although the overall rates of immune checkpoint inhibitor–associated edema found in this study exceed that of Velev et al.,^14^ rates of grade 3-5 edema for treatment-related , any-cause, and immune-related peripheral edema at 0.18% (*n* = 23 of 12 962), 0.23% (*n* = 21 of 9123), and 0% (*n* = 0 of 505), respectively, are more consistent with their findings. This is logical as the data from Velev et al. were extracted from the Gustave Roussy Cancer Center’s “Registry of Severe Adverse Reactions to Immunomodulatory Monoclonal Antibodies used in Oncology (REISAMIC)”^14^ and thus less extreme presentations may have been excluded from the database.

Our findings further suggest that immune checkpoint inhibitor monotherapy is associated with a lower incidence rate of any grade treatment-related peripheral edema relative to taxane chemotherapy and that the overall add-on effect of immune checkpoint inhibitors on peripheral edema risk when combined with systemic therapy is not statistically significant. Our aggregate incidence analysis demonstrated that nonimmune checkpoint inhibitor–containing regimens were associated with a grade 1-5 treatment-related peripheral edema incidence of 6.2% (*n* = 340 of 5482) compared with 4.0% (*n* = 1034 of 25 579) in immune checkpoint inhibitor–containing regimens. Consistent with these results, Tian et al.^15^ reported across 27 phase 2-3 PD-1 and PD-L1 RCTs that PD-1 and PD-L1 inhibitors were associated with a statistically lower risk of all-grade peripheral edema when evaluated against chemotherapy alone. In addition, no statistically significant difference in all-grade peripheral edema was observed in their meta-analyses when PD-1 or PD-L1 plus chemotherapy regimens were compared with chemotherapy alone.^15^ While arriving at similar conclusions, our results are more nuanced secondary to subsetting the analysis by adverse event modality and granular treatment regimens.

Reported incidences of grade 3 or higher peripheral edema with single-agent taxanes has been reported at 0.3%-0.5% for paclitaxel and 2.8%-12.6% for docetaxel,^16^ both substantially higher than the 0.1%-0.2% observed with immune checkpoint inhibitor monotherapy in this study. Taken together, our findings suggest that immune checkpoint inhibitors likely contribute less peripheral edema risk than taxane-containing regimens.^17^^,^^18^ This has important clinical implications for the management of edema in patients receiving approved immune checkpoint inhibitor–taxane combinations, such as in triple-negative breast cancer^19^ or non-small cell lung cancer,^20^ and may support continuation of immune checkpoint inhibitors with initial discontinuation of taxane therapy in such cases.^15^ By contrast, the present study did not demonstrate statistically significant differences in the incidence of peripheral edema between immune checkpoint inhibitors and nontaxane chemotherapy. Such combinations are currently approved across multiple malignancies including gastric,^21^ urothelial,^22^ breast,^23^ and lung cancers (both non-small and small cell).^24^^,^^25^ In patients who develop edema during treatment with immune checkpoint inhibitors plus nontaxane chemotherapy, our findings suggest that discontinuation of either agent may be a reasonable management approach. Our study-level meta-regression analysis showed grade 1-5 treatment-related edema and follow-up duration are jointly, but not independently, associated with treatment-related discontinuation. Therefore, edema is unlikely to be the sole driver for stopping immune checkpoint inhibitor therapy, but rather discontinuation patterns likely reflect a combined burden of follow-up time and multiple treatment-related toxicities.

As a rare and recently defined phenomenon, management strategies for immune-related peripheral edema remain unestablished. Managing immune-related edema first requires ruling out nonimmunogenic causes of peripheral edema such as heart failure or acute kidney injury, and ideally determining if the edema is secondary to another immune-related adverse event before intervention.^14^^,^^26^ Similar to relatively rare immune-related adverse events such as pancreatitis and pericarditis, the primary intervention appears to be high-dose glucocorticoid steroids with occasional use of immunosuppressants in addition to a temporary vs permanent discontinuation of immune checkpoint inhibitor therapy.^14^^,^^15^^,^^27-29^ In our literature review of the treatment outcomes for immune-related peripheral edema, primary immune-related edema appears to require greater rates of high dose steroids and interruption in immune checkpoint inhibitor therapy when compared with secondary immune-related edema. Further studies elucidating the pathophysiology of immune-related edema such as its relationship to endothelial dysfunction with sinusoidal obstruction syndrome/veno-occlusive disease or capillary leak syndrome^14^ are needed to delineate evidence-based management.

Several limitations of this study warrant consideration. Foremost, adverse event identification and classification (eg, treatment-related adverse event, immune-related adverse event, any-cause) rely heavily on the judgment of individual clinicians. Given that edema is a relatively newly recognized immune-related adverse event, reporting may be inconsistent. Studies such as Hsiehchen et al.^30^ have reported poor concordance of interrater agreement of the occurrence and severity of common immune-related adverse events such as adrenal insufficiency, colitis, hepatitis, and pneumonitis. Xie et al.^31^ found great inconsistency and inadequacy in immune-related adverse event reporting in PD-1 and PD-L1 inhibitors, with definitions changing over time and different definitions being used among separate immune checkpoint inhibitor classes. Thus, misclassification of adverse events as treatment-related or any-cause adverse events may contribute to the underreporting of immune-related adverse events in this study, especially grade 3-5 edema, leading to a limited sample size. Likewise, not all RCTs evaluating immune checkpoint inhibitors reported peripheral edema, possibly because its significance as a potential immune-related adverse event was not fully recognized.^9^ American Society of Clinical Oncology Practice and Society for Immunotherapy of Cancer Guidelines outline management of peripheral edema secondary to immune-related cardio or renal toxicity,^3^^,^^26^ however no guidelines exist for primary immune-related peripheral edema. This likely influences the underrecognition and underreporting of immune-related peripheral edema. Of note, CTCAE-based reporting of treatment-related adverse events in combination therapy trials does not require attribution of events to a specific agent. As a result, treatment-related edema occurring in patients receiving immune checkpoint inhibitor–containing combination regimens cannot be definitively classified as primary (immune-related) vs secondary (because of systemic therapy such as chemotherapy, molecular-targeted therapy, or other causes). Similarly, most included studies did not specify whether discontinuations were directly attributable to a specific adverse event, limiting the ability to isolate edema-specific effects. Demographic information on individual patients was not available for the meta-analysis of edema incidence across adverse event classifications and therapy modalities; future analysis is needed to explore the role of clinical characteristics in immune checkpoint inhibitor–related peripheral edema. Finally, in this study, clinical subtypes such as sinusoidal-obstruction-syndrome or capillary leak syndrome were not explored, limiting the granularity of mechanism exploration.

Peripheral edema is a rare but potentially serious adverse event associated with immune checkpoint inhibitors. Immune checkpoint inhibitors likely contribute less peripheral edema risk than taxane therapy, and the overall add-on effect of immune checkpoint inhibitors on peripheral edema risk when combined with systemic therapy is not statistically significant. However, immune checkpoint inhibitor–related peripheral edema may lead to immune checkpoint inhibitor treatment interruption and/or discontinuation, perhaps unnecessarily at times, particularly in the combination treatment setting. Although infrequent, the clinical uncertainty surrounding immune checkpoint inhibitor–associated edema may drive avoidable treatment interruptions, reinforcing the need for mechanistic studies and standardized management approaches.

## Supplementary Material

pkag058_Supplementary_Data

## Data Availability

All data are incorporated into the article and its online supplementary material.
