# Supplementary material for: Incidence and treatment-modifying impact of immune checkpoint inhibitor–associated peripheral edema: a systematic review and meta-analysis
Source: JNCI Cancer Spectr. 2026 Jun 3;10(4):pkag058. doi: 10.1093/jncics/pkag058 (PMC13428319; doi:10.1093/jncics/pkag058)
Supplement: pkag058_Supplementary_Data [file pkag058_supplementary_data.docx]

**Methods S1.** Supplementary Methods

The following data was extracted from each eligible study: PMID, first author, study name, cancer type/subtype, treatment setting (Palliative, Adjuvant, Neoadjuvant, Definitive), blindness status (OL/DB), ICI subtype(s) (PD-1, PD-L1, CTLA-4), study design (ICI +/- Y vs X), therapeutic regimens for comparison, number of patients in each treatment arm, AE outcome class (TRAE, Any Cause, irAE), number of any-grade and grade 3-5 AE edema occurrences. The severity of AEs was in accordance with the Common Terminology Criteria for Adverse Events (CTCAE) is shown below. Cochrane risk of bias excel tool for randomized trials (RoB 2) was utilized.

| CTCAE Term = **Generalized edema** | Grade 1 | Grade 2 | Grade 3 | Grade 4 | Grade 5 |
| --- | --- | --- | --- | --- | --- |
| A disorder characterized by fluid accumulation in the tissues of the body including the skin | Noted on exam; 1+ pitting  edema | Interfering with instrumental  ADLs; oral therapy initiated | Interferes with self care ADL;  intravenous therapy  indicated; skin breakdown | Life-threatening consequences | - |

*CTCAE Version 5.0*

| CTCAE Term =  **Edema limbs** | Grade 1 | Grade 2 | Grade 3 | Grade 4 | Grade 5 |
| --- | --- | --- | --- | --- | --- |
| A disorder characterized by swelling due to excessive fluid accumulation in the upper or lower extremities. | 5 - 10% inter-limb discrepancy in volume or circumference at point of greatest visible difference; swelling or obscuration of anatomic architecture on close inspection | >10 - 30% inter-limb  discrepancy in volume or  circumference at point of  greatest visible difference;  readily apparent obscuration of anatomic architecture; obliteration of skin folds; readily apparent deviation from normal anatomic contour; limiting instrumental ADL | >30% inter-limb discrepancy in volume; gross deviation from normal anatomic contour; limiting self care ADL | - | - |

*CTCAE Version 4.0*

[ADL: activities of daily living, AE: adverse event, Chemo: chemotherapy, CTLA-4: cytotoxic T-lymphocyte antigen-4, ICI: immune checkpoint inhibitor, irAE: immune-related adverse event, Mono: Monotherapy, MTT: molecular targeted therapy, PD-1: programmed death protein-1, PD-L1: programmed death-ligand-1, TRAE: treatment related adverse event]

**Table S1.** Search strategy for systematic review of the incidence of peripheral edema

| **Tools** | **Search Words** |
| --- | --- |
| **Medline** | (“Atezolizumab”[All Fields] OR “Avelumab”[All Fields] OR “Cemiplimab”[All Fields] OR “Durvalumab”[All Fields] OR “Ipilimumab”[All Fields] OR “Nivolumab”[All Fields] OR “Pembrolizumab”[All Fields] OR “Tremelimumab”[All Fields] OR “Tecentriq”[All Fields] OR “Bavencio”[All Fields] OR “Libtayo”[All Fields] OR “Imfinzi”[All Fields] OR “Yervoy”[All Fields] OR “Opdivo”[All Fields] OR “Keytruda”[All Fields] OR “Spartalizumab”[All Fields] OR “Immune checkpoint inhibitor”[All Fields] OR “Immune checkpoint blockade”[All Fields] OR “MPDL3280A”[All Fields] OR “RG7446”[All Fields] OR “MSB0010718C”[All Fields] OR “REGN2810”[All Fields] OR “MEDI4736”[All Fields] OR “BMS-734016”[All Fields] OR “MDX-010”[All Fields] OR “MDX-101”[All Fields] OR “ONO-4538”[All Fields] OR “BMS-936558”[All Fields] OR “MDX1106”[All Fields] OR “MK-3475”[All Fields] OR “lambrolizumab”[All Fields] OR “CP-675,206”[All Fields] OR “Ticilimumab”[All Fields] OR “PDR001”[All Fields] OR “PD-1”[All Fields] OR “PD-L1”[All Fields] or “CTLA-4”[All Fields] OR “SHR-1210”[All Fields] OR “ICIs”[All Fields] OR “camrelizumab”[All Fields] or “tislelizumab”[All Fields] OR “penpulimab”[All Fields] OR “toripalimab”[All Fields] OR “BGB-A317”[All Fields] OR “AK105”[All Fields] OR “JS001”[All Fields] OR “relatlimab”[All Fields] OR “LAG-3”[All Fields] OR “Opdualag”[All Fields] OR “Sintilimab”[All Fields] OR “Tyvyt”[All Fields]) AND (“Randomized controlled trial”[All Fields] OR “Randomized control trial”[All Fields] OR “RCT”[All Fields]) AND (“phase III”[All Fields] OR “phase 3”[All Fields] OR “phaseIII”[All Fields] OR “phase3”[All Fields] OR “phase-III”[All Fields] OR “phase-3”[All Fields] OR “P3”[All Fields]) AND (“neoplasm”[All Fields] OR “cancer”[All Fields] OR “malignancy”[All Fields] OR “tumor”[All Fields]) |
| **Embase** | (Atezolizumab OR Avelumab OR Cemiplimab OR Durvalumab OR Ipilimumab OR Nivolumab OR Pembrolizumab OR Tremelimumab OR ‘Immune checkpoint inhibitor’ OR Tecentriq OR Bavencio OR Libtayo OR Imfinzi OR Yervoy OR Opdivo OR Keytruda OR Spartalizumab OR ‘Immune checkpoint blockade’ OR MPDL3280A OR RG7446 OR MSB0010718C OR REGN2810 OR MEDI4736 OR BMS-734016 OR MDX-010 OR MDX-101 OR ONO-4538 OR BMS-936558 OR MDX1106 OR MK-3475 OR lambrolizumab OR CP-675,206 OR Ticilimumab OR PDR001 OR PD-1 OR PD-L1 or CTLA-4 OR SHR-1210 OR ICIs OR camrelizumab or tislelizumab OR penpulimab OR toripalimab OR BGB-A317 OR AK105 OR JS001 OR relatlimab OR LAG-3 OR Opdualag OR Sintilimab OR Tyvyt) AND (‘Randomized controlled trial’ OR ‘Randomized control trial’ OR RCT) AND (‘phase III’ OR ‘phase 3’ OR phaseIII OR phase3 OR phase-III OR phase-3 OR P3) AND (neoplasm OR cancer OR malignancy OR tumor) |
| **Web of Science** | #1 (Atezolizumab) OR (Avelumab) OR (Cemiplimab) OR (Durvalumab) OR (Ipilimumab) OR (Nivolumab) OR (Pembrolizumab) OR (Tremelimumab) OR (Immune checkpoint inhibitor) OR (Tecentriq) OR (Bavencio) OR (Libtayo) OR (Imfinzi) OR (Yervoy) OR (Opdivo) OR (Keytruda) OR (Spartalizumab) OR (Immune checkpoint inhibitor) OR (Immune checkpoint blockade) OR (MPDL3280A) OR (RG7446) OR (MSB0010718C) OR (REGN2810) OR (MEDI4736) OR (BMS-734016) OR (MDX-010) OR (MDX-101) OR (ONO-4538) OR (BMS-936558) OR (MDX1106) OR (MK-3475) OR (lambrolizumab) OR (CP-675,206) OR (Ticilimumab) OR (PDR001) OR (PD-1) OR (PD-L1) or (CTLA-4) OR (SHR-1210) OR (ICIs) OR (camrelizumab) or (tislelizumab) OR (penpulimab) OR (toripalimab) OR (BGB-A317) OR (AK105) OR (JS001) OR (relatlimab) OR (LAG-3) OR (Opdualag) OR (Sintilimab) OR (Tyvyt)  #2 (Randomized) OR (Randomised) OR (RCT) OR (Randomly)  #3 (phase III) OR (phase 3) OR (phaseIII) OR (phase3) OR (phase-III) OR (phase-3) OR (P3)  #4 (neoplasm) OR (cancer) OR (malignancy) OR (tumor)  #5 #1 AND #2 AND #3 AND #4 |

The last date for the literature search is February 27, 2023.

**Table S2.** Search strategy for systematic review of treatment of ICI related IRAE peripheral edema

| **Tools** | **Search Words** |
| --- | --- |
| **Medline** | (“edema”[All Fields] OR “capillary leak syndrome”[All Fields] OR “Sinusoidal obstruction syndrome”[All Fields] OR “veno-occlusive disease”[All Fields] OR “hydrops”[All Fields] OR “anasarca”[All Fields] OR “polyserositis”[All Fields] OR “serositis”[All Fields] OR “pleural effusion”[All Fields] OR “ascites”[All Fields] OR “pericardial effusion”[All Fields] OR “peritoneal effusion”[All Fields]) AND (“Atezolizumab”[All Fields] OR “Avelumab”[All Fields] OR “Cemiplimab”[All Fields] OR “Durvalumab”[All Fields] OR “Ipilimumab”[All Fields] OR “Nivolumab”[All Fields] OR “Pembrolizumab”[All Fields] OR “Tremelimumab”[All Fields] OR “Tecentriq”[All Fields] OR “Bavencio”[All Fields] OR “Libtayo”[All Fields] OR “Imfinzi”[All Fields] OR “Yervoy”[All Fields] OR “Opdivo”[All Fields] OR “Keytruda”[All Fields] OR “Spartalizumab”[All Fields] OR “Immune checkpoint inhibitor”[All Fields] OR “Immune checkpoint blockade”[All Fields] OR “MPDL3280A”[All Fields] OR “RG7446”[All Fields] OR “MSB0010718C”[All Fields] OR “REGN2810”[All Fields] OR “MEDI4736”[All Fields] OR “BMS-734016”[All Fields] OR “MDX-010”[All Fields] OR “MDX-101”[All Fields] OR “ONO-4538”[All Fields] OR “BMS-936558”[All Fields] OR “MDX1106”[All Fields] OR “MK-3475”[All Fields] OR “lambrolizumab”[All Fields] OR “CP-675,206”[All Fields] OR “Ticilimumab”[All Fields] OR “PDR001”[All Fields] OR “PD-1”[All Fields] OR “PD-L1”[All Fields] or “CTLA-4”[All Fields] OR “SHR-1210”[All Fields] OR “ICIs”[All Fields] OR “camrelizumab”[All Fields] or “tislelizumab”[All Fields] OR “penpulimab”[All Fields] OR “toripalimab”[All Fields] OR “BGB-A317”[All Fields] OR “AK105”[All Fields] OR “JS001”[All Fields] OR “relatlimab”[All Fields] OR “LAG-3”[All Fields] OR “Opdualag”[All Fields] OR “Sintilimab”[All Fields] OR “Tyvyt”[All Fields]) AND (“neoplasm”[All Fields] OR “cancer”[All Fields] OR “malignancy”[All Fields] OR “tumor”[All Fields]) AND (“adverse event”[All Fields] OR “side effect”[All Fields] OR “immune-related adverse event”[All Fields] OR “treatment-related adverse event”[All Fields]) AND (“treatment”[All Fields] OR “therapy”[All Fields] OR “management”[All Fields]) |
| **Embase** | (edema OR ‘capillary leak syndrome’ OR ‘Sinusoidal obstruction syndrome’ OR ‘veno-occlusive disease’ OR hydrops OR anasarca OR polyserositis OR serositis OR ‘pleural effusion’ OR ascites OR ‘pericardial effusion’ OR ‘peritoneal effusion’) AND (Atezolizumab OR Avelumab OR Cemiplimab OR Durvalumab OR Ipilimumab OR Nivolumab OR Pembrolizumab OR Tremelimumab OR ‘Immune checkpoint inhibitor’ OR Tecentriq OR Bavencio OR Libtayo OR Imfinzi OR Yervoy OR Opdivo OR Keytruda OR Spartalizumab OR ‘Immune checkpoint blockade’ OR MPDL3280A OR RG7446 OR MSB0010718C OR REGN2810 OR MEDI4736 OR BMS-734016 OR MDX-010 OR MDX-101 OR ONO-4538 OR BMS-936558 OR MDX1106 OR MK-3475 OR lambrolizumab OR CP-675,206 OR Ticilimumab OR PDR001 OR PD-1 OR PD-L1 or CTLA-4 OR SHR-1210 OR ICIs OR camrelizumab or tislelizumab OR penpulimab OR toripalimab OR BGB-A317 OR AK105 OR JS001 OR relatlimab OR LAG-3 OR Opdualag OR Sintilimab OR Tyvyt) AND (neoplasm OR cancer OR malignancy OR tumor) AND (‘adverse event’ OR ‘side effect’ OR ‘immune-related adverse event’ OR ‘treatment-related adverse event’) AND (treatment OR therapy OR management) |
| **Web of Science** | #1 (edema) OR (capillary leak syndrome) OR (Sinusoidal obstruction syndrome) OR (veno-occlusive disease) OR (hydrops) OR (anasarca) OR (polyserositis) OR (serositis) OR (pleural effusion) OR (ascites) OR (pericardial effusion) OR (peritoneal effusion)  #2 (Atezolizumab) OR (Avelumab) OR (Cemiplimab) OR (Durvalumab) OR (Ipilimumab) OR (Nivolumab) OR (Pembrolizumab) OR (Tremelimumab) OR (Immune checkpoint inhibitor) OR (Tecentriq) OR (Bavencio) OR (Libtayo) OR (Imfinzi) OR (Yervoy) OR (Opdivo) OR (Keytruda) OR (Spartalizumab) OR (Immune checkpoint inhibitor) OR (Immune checkpoint blockade) OR (MPDL3280A) OR (RG7446) OR (MSB0010718C) OR (REGN2810) OR (MEDI4736) OR (BMS-734016) OR (MDX-010) OR (MDX-101) OR (ONO-4538) OR (BMS-936558) OR (MDX1106) OR (MK-3475) OR (lambrolizumab) OR (CP-675,206) OR (Ticilimumab) OR (PDR001) OR (PD-1) OR (PD-L1) or (CTLA-4) OR (SHR-1210) OR (ICIs) OR (camrelizumab) or (tislelizumab) OR (penpulimab) OR (toripalimab) OR (BGB-A317) OR (AK105) OR (JS001) OR (relatlimab) OR (LAG-3) OR (Opdualag) OR (Sintilimab) OR (Tyvyt)  #3 (neoplasm) OR (cancer) OR (malignancy) OR (tumor)  #4 (adverse event) OR (side effect) OR (immune-related adverse event) OR (treatment-related adverse event)  #5 (treatment) or (therapy) or (management)  #4 #1 AND #2 AND #3 AND #4 AND #5 |

The last date for the literature search is February 27, 2023.

**Table S3.**

1. **Inclusion Criteria for Search Strategy for Systematic Review of the Incidence of Peripheral Edema**

| **Inclusion Criteria** |  |
| --- | --- |
| **1** | Phase 3 RCT |
| **2** | At least 1 arm of the clinical trial evaluates at least 1 ICI with/without other systematic therapy |
| **3** | Patients were clinically diagnosed with any type of solid tumor |
| **4** | Study includes any grade adverse events (AEs) or grade 3-5 AEs of peripheral edema |
| **5** | Full text papers |

| **Exclusion Criteria** |  |
| --- | --- |
| **1** | Narrative review, systematic review, meta-analysis, cohort studies, case series/reports |
| **2** | Conference abstracts |
| **3** | Studies published in languages other than English |
| **4** | Studies with unclarity in exact treatment regimen in each arm |

1. **Inclusion Criteria for Search Strategy for Systematic Review of Treatment of ICI Related IRAE Peripheral Edema**

| **Inclusion Criteria** |  |
| --- | --- |
| **1** | Published study reporting peripheral edema among patients treated with systemic therapy including ICI |
| **2** | Clinical studies including RCT, prospective clinical trials, retrospective trials, case series, and case reports |
| **3** | Patients were clinically diagnosed with any type of solid tumor |
| **4** | Study includes any grade immune related adverse events (irAEs) or grade 3-5 irAEs of peripheral edema |
| **5** | Full text papers |

| **Exclusion Criteria** |  |
| --- | --- |
| **1** | Narrative review, systematic review, meta-analysis |
| **2** | Conference abstracts |
| **3** | Studies published in languages other than English |
| **4** | Studies with unclarity in exact treatment regimen in each arm |

Abbreviations [AE: adverse event, ICI: immune checkpoint inhibitor, irAE: immune-related adverse event, RCT: randomized controlled trial]

**Table S4.** Characteristics of studies included in meta-analysis for incidence

| **PMID** | **Last Name, Year** | **Study Name** | **Cancer** | **Setting** | **OL/DB** | **ICI subtype** | **Study Design** | **Outcome** |
| --- | --- | --- | --- | --- | --- | --- | --- | --- |
| 23295794 | Ribas 2013 | NCT00257205 | Melanoma | Pal | OL | CTLA4 | ICI vs chemo | TRAE |
| 25891173 | Robert 2015 | KEYNOTE-006 | Melanoma | Pal | OL | PD-1, CTLA4 | ICI vs ICI vs ICI | Any-cause |
| 26028407 | Brahmer 2015 | CheckMate 017 | NSCLC | Pal | OL | PD-1 | ICI vs chemo | TRAE |
| 26406148 | Motzer 2015 | CheckMate 025 | RCC | Pal | OL | PD-1 | ICI vs MT | TRAE |
| 26412456 | Borghaei 2015 | CheckMate 057 | NSCLC | Pal | OL | PD-1 | ICI vs chemo | TRAE |
| 26712084 | Herbst 2016 | KEYNOTE-010 | NSCLC | Pal | OL | PD-1 | ICI vs ICI vs chemo | TRAE |
| 27979383 | Rittmeyer 2017 | OAK | NSCLC | Pal | OL | PD-L1 | ICI vs chemo | TRAE |
| 28212060 | Bellmunt 2017 | KEYNOTE-045 | RCC | Pal | OL | PD-1 | ICI vs chemo | Any-cause |
| 28993052 | Kang 2017 | ATTRACTION-2 | Gastric or GEJ | Pal | DB | PD-1 | ICI vs placebo | Any-cause |
| 29658856 | Gandhi 2018 | KEYNOTE-189 | NSCLC | Pal | DB | PD-1 | ICI + chemo vs placebo + chemo | Any-cause |
| 30262187 | Barlesi 2018 | JAVELIN Lung 200 | NSCLC | Pal | OL | PD-L1 | ICI vs chemo | TRAE |
| 30280641 | Horn 2018 | IMpower 133 | SCLC | Pal | DB | PD-L1 | ICI + chemo vs placebo + chemo | TRAE |
| 30345906 | Schmid 2018 | IMpassion 130 | Breast | Pal | DB | PD-L1 | ICI + chemo vs placebo + chemo | Any-cause |
| 30361170 | Hodi 2018 | CheckMate 067 | Melanoma | Pal | DB | PD-1, CTLA4 | Dual ICI vs ICI vs ICI | TRAE |
| 30509740 | Cohen 2019 | KEYNOTE-040 | HNSCC | Pal | OL | PD-1 | ICI vs chemo | TRAE |
| 30659987 | Wu 2019 | CheckMate 089 | NSCLC | Pal | OL | PD-1 | ICI vs chemo | TRAE |
| 30779531 | Motzer 2019 | JAVELIN Renal 101 | RCC | Pal | OL | PD-L1 | ICI + MT vs MT | TRAE |
| 31003911 | Eng 2019 | IMblaze370 | CRC | Pal | OL | PD-L1 | ICI + MT vs ICI vs MT | Any-cause |
| 31050707 | Fradet 2019 | KEYNOTE-045 | Urothelial | Pal | OL | PD-1 | ICI vs chemo | TRAE |
| 31122901 | West 2019 | IMpower130 | NSCLC | Pal | OL | PD-L1 | ICI + chemo vs chemo | Any-cause |
| 31221619 | Long 2019 | KEYNOTE-252 | Melanoma | Pal | DB | PD-1 | ICI + MT vs ICI + placebo | TRAE |
| 31786121 | Schmid 2020 | IMpassion130 | Breast | Pal | DB | PD-L1 | ICI + chemo vs placebo + chemo | Any-cause |
| 31790344 | Finn 2020 | KEYNOTE-240 | HCC | Pal | DB | PD-1 | ICI vs Placebo | Any-cause |
| 32271377 | Rizvi 2020 | MYSTIC | NSCLC | Pal | OL | PD-L1, CTLA4 | ICI vs dual ICI vs chemo | TRAE |
| 32468956 | Rudin 2020 | KEYNOTE-604 | SCLC | Pal | DB | PD-1 | ICI + chemo vs chemo | Any-cause |
| 32534646 | Gutzmer 2020 | IMspire150 | Melanoma | Pal | DB | PD-L1 | ICI + MT vs Placebo + MT | TRAE |
| 32966830 | Mittendorf 2020 | IMpassion031 | Breast | Neo | DB | PD-L1 | ICI + chemo vs placebo + chemo | Any-cause |
| 32997575 | Robert 2020 | CheckMate 066 | Melanoma | Pal | DB | PD-1 | ICI + placebo vs placebo + chemo | TRAE |
| 33285097 | Goldman 2020 | CASPIAN | SCLC | Pal | OL | PD-L1. CTLA4 | dual ICI + chemo vs ICI + chemo vs chemo | Any-cause |
| 33309774 | Gogas 2021 | IMspire 170 | Melanoma | Pal | OL | PD-1, PD-L1 | ICI + MT vs ICI | Any-cause |
| 33347829 | Zhou 2021 | CameL | NSCLC | Pal | OL | PD-1 | ICI + chemo vs chemo | TRAE |
| 33476593 | Paz-Ares 2021 | CheckMate 9LA | NSCLC | Pal | OL | PD-1, CTLA4 | Dual ICI + chemo vs chemo | TRAE |
| 33616314 | Motzer 2021 | CLEAR | RCC | Pal | OL | PD-1 | ICI + MT vs MT + MT vs MT | TRAE |
| 33657295 | Choueiri 2021 | CheckMate 9ER | RCC | Pal | OL | PD-1, CTLA4 | ICI + MT vs MT vs Dual ICI + MT | TRAE |
| 33794205 | Lee 2021 | JAVELIN Head and Neck 100 | HNSCC | Pal | DB | PD-L1 | ICI + CRT vs Placebo vs CRT | TRAE |
| 33894335 | Rodriguez-Abreu 2021 | KEYNOTE-189 | NSCLC | Pal | DB | PD-1 | ICI + chemo vs placebo + chemo | Any-cause |
| 34051178 | Powles 2021 | KEYNOTE-361 | Urothelial | Pal | OL | PD-1 | ICI + chemo vs ICI vs chemo | TRAE |
| 34143970 | Pujade-Lauraine 2021 | JAVELIN Ovarian 200 | Ovarian | Pal | OL | PD-L1 | ICI + chemo vs chemo vs ICI | TRAE |
| 34143979 | Pusztai 2021 | I-SPY2 | Breast | Neo | OL | PD-L1 | ICI + MT + chemo vs chemo | irAE |
| 34219000 | Miles 2021 | IMpassion131 | Breast | Pal | DB | PD-L1 | ICI + chemo vs placebo + chemo | irAE |
| 34272041 | Emens 2021 | IMpassion130 | Breast | Pal | DB | PD-L1 | ICI + chemo vs placebo + chemo | Any-cause |
| 34363762 | Monk 2021 | JAVELIN Ovarian 100 | Ovarian | Def | OL | PD-L1 | Chemo followed by ICI vs ICI + chemo followed by ICI vs chemo followed by observation | TRAE |
| 34555333 | Felip 2021 | IMpower010 | NSCLC | Adj | OL | PD-L1 | ICI vs Observation | Any-cause |
| 34656227 | Fennell 2021 | CONFIRM | Mesothelioma | Pal | DB | PD-1 | ICI vs placebo | TRAE |
| 35030011 | Dummer 2022 | COMBI-i | Melanoma | Pal | DB | PD-1 | ICI + MT vs placebo + MT | Any-cause |
| 35139273 | Tewari 2022 | EMPOWER-Cervical 1/GOG-3016/ENGOT-cx9 | Cervical | Pal | OL | PD-1 | ICI vs chemo | Any-cause |
| 35139274 | Schmid 2022 | KEYNOTE-522 | Breast | Neo | DB | PD-1 | ICI + chemo vs placebo + chemo | Any-cause |
| 35688173 | Motzer 2022 | CheckMate 9ER | RCC | Pal | OL | PD-1 | ICI + MT vs MT | Any-cause |
| 35798016 | Kelley 2022 | COSMIC-312 | HCC | Pal | OL | PD-L1 | ICI + MT vs MT vs MT | TRAE |
| 35998300 | Chesney 2023 | MASTERKEY-265 | Melanoma | Pal | DB | PD-1 | ICI + vaccine vs ICI + placebo | TRAE |
| 36008722 | Gogishvili 2022 | EMPOWER-Lung 3 | NSCLC | Pal | DB | PD-1 | ICI + chemo vs placebo + chemo | TRAE |
| 36099926 | Pal 2022 | IMmotion010 | RCC | Adj | DB | PD-L1 | ICI vs placebo | Any-cause |
| 36108662 | O'Brien 2022 | PEARLS/KEYNOTE-091 | NSCLC | Adj | DB | PD-1 | ICI vs placebo | TRAE |
| 36166727 | Atkins 2022 | DREAMseq | Melanoma | Pal | OL | PD-1. CTLA4 | dual ICI vs MT + MT | Any-cause |
| 36369901 | Yoshino 2023 | KEYNOTE-177 | CRC | Pal | OL | PD-1 | ICI vs chemo | TRAE |
| 36460017 | Ascierto 2023 | IMspire150 | Melanoma | Pal | DB | PD-L1 | ICI + MT vs MT | TRAE |
| 36477031 | Rohaan 2022 | NCT02278887 | Melanoma | Pal | OL | CTLA4 | Cellular therapy vs ICI | TRAE |
| 32201234 | Planchard 2020 | ARCTIC B | NSCLC | Pal | OL | PD-L1, CTLA4 | Dual ICI vs chemo vs ICI vs IVI | Any-cause |

(Continued)

| **PMID** | **Treatment (T1)** | **T2** | **T3** | **T4** | **N (T1)** | **N (T2)** | **N (T3)** | **N (T4)** | **Taxan (DTX, PTX)** |
| --- | --- | --- | --- | --- | --- | --- | --- | --- | --- |
| 23295794 | Trem | DTIC or TMZ | - | - | 325 | 319 | - | - | Non Taxan |
| 25891173 | Pem (q2 weeks) | Pem (q3 weeks) | Ipi | - | 278 | 277 | 256 | - | - |
| 26028407 | Nivo | DTX | - | - | 131 | 129 | - | - | Taxan |
| 26406148 | Nivo | Everolimus | - | - | 406 | 397 | - | - | - |
| 26412456 | Nivo | DTX | - | - | 287 | 268 | - | - | Taxan |
| 26712084 | Pem (2mg/kg) | Pem (10mg/kg) | DTX | - | 339 | 343 | 309 | - | Taxan |
| 27979383 | Atezo | DTX | - | - | 607 | 578 | - | - | Taxan |
| 28212060 | Pem | PTX, DTX, or Vinflunine | - | - | 266 | 255 | - | - | Mix |
| 28993052 | Nivo | placebo | - | - | 330 | 161 | - | - | - |
| 29658856 | Pem + Cis+Carb + PMTX | Placebo + Cis+Carbo + PMTX | - | - | 405 | 202 | - | - | Non Taxan |
| 30262187 | Ave | DTX | - | - | 393 | 365 | - | - | Taxan |
| 30280641 | Atezo + Carbo + VP-16 | Placebo + Carbo + VP-16 | - | - | 198 | 196 | - | - | Non Taxan |
| 30345906 | Atezo+ PTX | Placebo + PTX | - | - | 452 | 438 | - | - | Taxan |
| 30361170 | Nivo + Ipi | Nivo | Ipi | - | 313 | 313 | 311 | - | - |
| 30509740 | Pem | MTX, DTX, or cetuximab | - | - | 246 | 234 | - | - | Mix |
| 30659987 | Nivo | DTX | - | - | 337 | 156 | - | - | Taxan |
| 30779531 | Ave + Axitinib | Sunitinib | - | - | 434 | 439 | - | - | - |
| 31003911 | Atezo + Cobimetinib | Atezo | Regorafenib | - | 179 | 90 | 80 | - | - |
| 31050707 | Pem | PTX, DTX, or Vinflunine | - | - | 266 | 255 | - | - | Mix |
| 31122901 | Atezo + Carbo + PTX | Carbo + PTX | - | - | 473 | 232 | - | - | Mix |
| 31221619 | Pem + Epacadostat | Pem + Placebo | - | - | 353 | 352 | - | - | - |
| 31786121 | Atezo + PTX | Placebo + PTX | - | - | 453 | 437 | - | - | Taxan |
| 31790344 | Pem | Placebo | - | - | 279 | 134 | - | - | - |
| 32271377 | Durv | Druv + Trem | PTX + Carbo, PMTX + Cis or Carbo, Gem + Cis or Carbo | - | 369 | 371 | 352 | - | Mix |
| 32468956 | Pem + Cis/Carbo + VP-16 | Placebo + Cis/Carbo + VP-16 | - | - | 223 | 223 | - | - | Non Taxan |
| 32534646 | Atezo + Cobimetinib + Vemurafenib | Placebo + Cobimetinib + Vemurafenib | - | - | 230 | 281 | - | - | - |
| 32966830 | Atezo + PTX + DOX + CP | Placebo + PTX + DOX + CP | - | - | 164 | 167 | - | - | Mix |
| 32997575 | Nivo + Placebo | Placebo + DTIC | - | - | 206 | 205 | - | - | Non Taxan |
| 33285097 | Durv + Trem + VP-16 + Carbo or Cis | Durv + VP-16 + Carbo or Cis | VP-16 + Carbo or Cis | - | 268 | 268 | 269 | - | Non Taxan |
| 33309774 | Atezo + Cobimetinib | Pem | - | - | 220 | 216 | - | - | - |
| 33347829 | Camr + Carbo + PMTX | Carbo + PMTX | - | - | 205 | 207 | - | - | Non Taxan |
| 33476593 | Nivolumab + ipilimumab + either (Carbo + PTX) or (Carbo + PMTX) or (Cis + PMTX) | Carbo + PTX, Carbo + PMTX, Cis + PMTX | - | - | 358 | 349 | - | - | Mix |
| 33616314 | Pem + Lenvatinib | Lenvatinib + Everolimus | Sunitinib | - | 352 | 355 | 340 | - | - |
| 33657295 | Nivo + Cabozantinib | Sunitinib | - | - | 320 | 320 | - | - | - |
| 33794205 | Ave + Cis + Radiotherapy | Placebo + Cis + Radiotherapy | - | - | 348 | 344 | - | - | Non Taxan |
| 33894335 | Pem + PMTX + Cis or Carbo | placebo + PMTX + Cis or Carbo | - | - | 405 | 202 | - | - | Non Taxan |
| 34051178 | Pem + Gem + Cis or Carbo | Pem | Gem + Cis or Carbo | - | 349 | 302 | 342 | - | Non Taxan |
| 34143970 | Ave + DOX | DOX | Ave | - | 188 | 190 | 188 | - | Non Taxan |
| 34143979 | Durv + olaparib + PTX | PTX | - | - | 73 | 299 | - | - | Taxan |
| 34219000 | Atezo + PTX | Placebo + PTX | - | - | 432 | 217 | - | - | Taxan |
| 34272041 | Atezo + PTX | placebo + PTX | - | - | 460 | 430 | - | - | Taxan |
| 34363762 | Carbo + PTX + Ave | Carbo + PTX + Ave + Ave (2nd dose) | Carbo + PTX | - | 328 | 329 | 334 | - | Mix |
| 34555333 | Atezo | Observation | - | - | 495 | 495 | - | - | - |
| 34656227 | Nivo | placebo | - | - | 221 | 111 | - | - | - |
| 35030011 | Spart + Dabrafenib + Trametinib | placebo + Dabrafenib + Trametinib | - | - | 267 | 264 | - | - | - |
| 35139273 | Cem | PMTX, topotecan, irinotecan, Gem, or VRL | - | - | 300 | 290 | - | - | Non Taxan |
| 35139274 | Pem + Placebo + Carbo + PTX + (DOX + CP) or (EPI + CP) | Placebo + Carbo + PTX + (DOX + CP) or (EPI + CP) | - | - | 783 | 389 | - | - | Mix |
| 35688173 | Nivo + Cabozantinib | Sunitinib | - | - | 320 | 320 | - | - | - |
| 35798016 | Atezo + Cabozantinib | Sorafenib | Cabozantinib | - | 429 | 207 | 188 | - | - |
| 35998300 | T-vec + Pem | Placebo + Pem | - | - | 345 | 343 | - | - | - |
| 36008722 | Cem + PTX + Carbo or Cis | Placebo + PTX + Carbo or Cis | - | - | 312 | 153 | - | - | Mix |
| 36099926 | Atezo | Placebo | - | - | 390 | 383 | - | - | - |
| 36108662 | Pem | Placebo | - | - | 580 | 581 | - | - | - |
| 36166727 | Nivo + Ipi followed by Dabrafenib + Trametinib | Dabrafenib + Trametinib followed by Nivo + Ipi | - | - | 126 | 130 | - | - | - |
| 36369901 | Pem | (FOLFOX6 or FOLFIRI) +/- (Bevacizumab or Cetuximab) | - | - | 22 | 25 | - | - | Non Taxan |
| 36460017 | Atezo + Cobimetinib + Vemurafenib | Placebo + Cobimetinib + Vemurafenib | - | - | 231 | 280 | - | - | - |
| 36477031 | TIL | Ipi | - | - | 80 | 82 | - | - | - |
| 32201234 | Durv + Trem | Erlotinib or GEM, or VNR | Durv | Trem | 173 | 110 | 117 | 60 | Non Taxan |

Abbreviations: [Atezo: Atezolizumab, Ave: Avelumab, Camr: Camrelizumab, Carb: Carboplatin, Cem: Cemiplimab, Chemo: chemotherapy, Cis: Cisplatin, CP: Cyclophosphamide, CRC: Colorectal cancer, CTLA-4: cytotoxic T-lymphocyte antigen-4, DTX: Docetaxel, DOX: Doxorubicin, DB: Double Blind, Durv: Durvalumab, EPI: Epirubicin, FOLFIRI: Irinotecan + Leucovorin + 5-fluoropyrimidine, FOLFOX6: Oxaliplatin + Leucovorin + 5-fluoropyrimidine, Camr: Camrelizumab, Gem: Gemcitabine, GEJ: Gastroesophageal junction, HCC: Hepatocellular carcinoma, HNSCC: Head and neck squamous cell cancer, ICI: immune checkpoint inhibitor, Ipi: Ipilimumab, irAE: immune-related adverse event, Mix: Taxan + X, X or Taxan, MTT: molecular targeted therapy, Nivo: Nivolumab, Non Taxan: chemo other than Taxan, NSCLC: Non-small cell lung cancer, OL: Open Label, PD-1: programmed death protein-1, PD-L1: programmed death-ligand-1, Pem: Pembrolizumab, PMTX: Pemetrexed, PTX: Paclitaxel, RCC: Renal cell cancer, SCLC: Small-cell cancer, Spart: Spartalizumab, Taxan: DTX, PTX, T-vec: Talimogene laherparepvec, TIL: Tumor-infiltrating lymphocytes, TMZ: temozolomide, TRAE: treatment related adverse event, Trem: Tremelimumab, VNR: Vinorelbine, VP-16: Etoposide, VRL: Vinorelbine, ー: MTT, Placebo, ICI]

**Table S5.A** Incidence of Grade 1-5 Edema – Total

| **AE** | **Therapy** | **Total** | | |
| --- | --- | --- | --- | --- |
|  |  | N | n (events) | Incidence |
| **TRAE**   **N = 12,962** | ICI (Mono) | 6969 | 195 | 2.80 |
|  | ICI (Dual) | 684 | 0 | 0 |
|  | ICI + Chemo | 2257 | 112 | 4.96 |
|  | ICI + MTT | 2349 | 205 | 8.73 |
|  | ICI + MTT + Chemo | 0 | 0 | - |
|  | Dual ICI + Chemo | 358 | 2 | 0.56 |
|  | ICI + Vaccine | 345 | 6 | 1.74 |
|  | Total | 12962 | 520 | 4.01 |
| **Any Cause**   **N = 9123** | ICI (Mono) | 3354 | 213 | 6.35 |
|  | ICI (Dual) | 429 | 34 | 7.93 |
|  | ICI + Chemo | 4086 | 550 | 13.46 |
|  | ICI + MTT | 986 | 142 | 14.40 |
|  | ICI + MTT + Chemo | 0 | 0 | - |
|  | Dual ICI + Chemo | 268 | 1 | 0.37 |
|  | ICI + Vaccine | 0 | 0 | - |
|  | Total | 9123 | 940 | 10.29 |
| **irAE**   **N = 505** | ICI (Mono) | 0 | 0 | - |
|  | ICI (Dual) | 0 | 0 | - |
|  | ICI + Chemo | 432 | 47 | 10.88 |
|  | ICI + MTT | 0 | 0 | - |
|  | ICI + MTT + Chemo | 73 | 15 | 20.55 |
|  | Dual ICI + Chemo | 0 | 0 | - |
|  | ICI + Vaccine | 0 | 0 | - |
|  | Total | 505 | 62 | 12.27 |

Abbreviations [AE: adverse event, Chemo: chemotherapy, ICI: immune checkpoint inhibitor, irAE: immune-related adverse event, Mono: Monotherapy, MTT: molecular targeted therapy, TRAE: treatment related adverse event]

**Table S5.A.1** Incidence of Grade 1-5 Edema – TRAE + irAE Combined

| **AE** | **Therapy** | **Total** | | |
| --- | --- | --- | --- | --- |
|  |  | N | n (events) | Incidence |
| **TRAE + irAE**  **N = 13,467** | ICI (Mono) | 6969 | 195 | 2.8 |
|  | ICI (Dual) | 684 | 0 | 0 |
|  | ICI + Chemo | 2689 | 159 | 5.91 |
|  | ICI + MTT | 2349 | 205 | 8.73 |
|  | ICI + MTT + Chemo | 73 | 15 | 20.55 |
|  | Dual ICI + Chemo | 358 | 2 | 0.56 |
|  | ICI + Vaccine | 345 | 6 | 1.74 |
|  | Total | 13,467 | 582 | 4.32 |

Abbreviations [AE: adverse event, Chemo: chemotherapy, ICI: immune checkpoint inhibitor, irAE: immune-related adverse event, Mono: Monotherapy, MTT: molecular targeted therapy, TRAE: treatment related adverse event]

**Table S5.B** Incidence of Grade 1-5 Edema - By ICI Type

| **AE** | **Therapy** | **PD-1** | | | **PD-L1** | | | **CTLA-4** | | | **PD-1 + CTLA4** | | | **PD-L1 + CTLA4** | | |
| --- | --- | --- | --- | --- | --- | --- | --- | --- | --- | --- | --- | --- | --- | --- | --- | --- |
|  |  | n | N | % | n | N | % | n | N | % | n | N | % | n | N | % |
| **TRAE**   **N = 12,962** | ICI (Mono) | 104 | 4694 | 2.22 | 58 | 1557 | 3.73 | 33 | 718 | 4.60 | 0 | 0 | - | 0 | 0 | - |
|  | ICI (Dual) | 0 | 0 | - | 0 | 0 | - | 0 | 0 | - | 0 | 313 | 0 | 0 | 371 | 0 |
|  | ICI + Chemo | 77 | 866 | 8.89 | 35 | 1391 | 2.52 | 0 | 0 | - | 0 | 0 | - | 0 | 0 | - |
|  | ICI + MTT | 52 | 1025 | 5.07 | 153 | 1324 | 11.56 | 0 | 0 | - | 0 | 0 | - | 0 | 0 | - |
|  | ICI + MTT + Chemo | 0 | 0 | - | 0 | 0 | - | 0 | 0 | - | 0 | 0 | - | 0 | 0 | - |
|  | Dual ICI + Chemo | 0 | 0 | - | 0 | 0 | - | 0 | 0 | - | 2 | 358 | 0.56 | 0 | 0 | - |
|  | ICI + Vaccine | 6 | 345 | 1.74 | 0 | 0 | - | 0 | 0 | - | 0 | 0 | - | 0 | 0 | - |
| **Any Cause**   **N = 9123** | ICI (Mono) | 124 | 1946 | 6.37 | 88 | 1092 | 8.06 | 1 | 316 | 0.32 | 0 | 0 | - | 0 | 0 | - |
|  | ICI (Dual) | 0 | 0 | - | 0 | 0 | - | 0 | 0 | - | 27 | 256 | 10.55 | 7 | 173 | 4.05 |
|  | ICI + Chemo | 266 | 1816 | 14.65 | 284 | 2270 | 12.51 | 0 | 0 | - | 0 | 0 | - | 0 | 0 | - |
|  | ICI + MTT | 72 | 587 | 12.27 | 70 | 399 | 17.54 | 0 | 0 | - | 0 | 0 | - | 0 | 0 | - |
|  | ICI + MTT + Chemo | 0 | 0 | - | 0 | 0 | - | 0 | 0 | - | 0 | 0 | - | 0 | 0 | - |
|  | Dual ICI + Chemo | 0 | 0 | - | 0 | 0 | - | 0 | 0 | - | 0 | 0 | - | 1 | 268 | 0.37 |
|  | ICI + Vaccine | 0 | 0 | - | 0 | 0 | - | 0 | 0 | - | 0 | 0 | - | 0 | 0 | - |
| **irAE**   **N = 505** | ICI (Mono) | 0 | 0 | - | 0 | 0 | - | 0 | 0 | - | 0 | 0 | - | 0 | 0 | - |
|  | ICI (Dual) | 0 | 0 | - | 0 | 0 | - | 0 | 0 | - | 0 | 0 | - | 0 | 0 | - |
|  | ICI + Chemo | 0 | 0 | - | 47 | 432 | 10.88 | 0 | 0 | - | 0 | 0 | - | 0 | 0 | - |
|  | ICI + MTT | 0 | 0 | - | 0 | 0 | - | 0 | 0 | - | 0 | 0 | - | 0 | 0 | - |
|  | ICI + MTT + Chemo | 0 | 0 | - | 15 | 73 | 20.55 | 0 | 0 | - | 0 | 0 | - | 0 | 0 | - |
|  | Dual ICI + Chemo | 0 | 0 | - | 0 | 0 | - | 0 | 0 | - | 0 | 0 | - | 0 | 0 | - |
|  | ICI + Vaccine | 0 | 0 | - | 0 | 0 | - | 0 | 0 | - | 0 | 0 | - | 0 | 0 | - |

Abbreviations: [AE: adverse event, Chemo: chemotherapy, CTLA-4: cytotoxic T-lymphocyte antigen-4, ICI: immune checkpoint inhibitor, irAE: immune-related adverse event, Mono: Monotherapy, MTT: molecular targeted therapy, PD-1: programmed death protein-1, PD-L1: programmed death-ligand-1, TRAE: treatment related adverse event]

**Table S5.C.1** Incidence of Grade 1-5 Edema – Primary vs Secondary Edema Comparison (Overall)

|  | Immune-related peripheral edema | | | Treatment-related peripheral edema | | | Any cause peripheral edema | | |
| --- | --- | --- | --- | --- | --- | --- | --- | --- | --- |
|  | Events | Total | % | Events | Total | % | Events | Total | % |
| Non-ICI Containing therapy | N/A | N/A | N/A | 340 | 5482 | 6.2 | 421 | 3375 | 12.47 |
| ICI containing therapy | 124 | 1010 | 12.28 | 1034 | 25579 | 4.04 | 1878 | 18246 | 10.29 |

**Table S5.C.2** Incidence of Grade 1-5 Edema – Primary vs Secondary Edema Comparison (Stratified by Therapy Class)

|  |  | Immune-related peripheral edema | | | Treatment-related peripheral edema | | | Any cause peripheral edema | | |
| --- | --- | --- | --- | --- | --- | --- | --- | --- | --- | --- |
|  | ICI Class | Events | Total | % | Events | Total | % | Events | Total | % |
| ICI (Mono) | Subtotal | N/A | N/A | N/A | 195 | 6969 | 2.8 | 213 | 3354 | 6.4 |
|  | PD-1 | N/A | N/A | N/A | 104 | 4694 | 2.2 | 124 | 1946 | 6.4 |
|  | PD-L1 | N/A | N/A | N/A | 58 | 1557 | 3.7 | 88 | 1092 | 8.1 |
|  | CTLA-4 | N/A | N/A | N/A | 33 | 718 | 4.6 | 1 | 316 | 0.3 |
| ICI (Dual) | Subtotal | N/A | N/A | N/A | 0 | 684 | 0 | 34 | 429 | 7.9 |
|  | PD-1 + CTLA-4 | N/A | N/A | N/A | 0 | 313 | 0 | 27 | 256 | 10.5 |
|  | PD-L1 + CTLA-4 | N/A | N/A | N/A | 0 | 371 | 0 | 7 | 173 | 4 |
| ICI + Chemo | Subtotal | 47 | 432 | 10.9 | 112 | 2257 | 5 | 549 | 4086 | 13.4 |
|  | PD-1 | N/A | N/A | N/A | 77 | 866 | 8.9 | 266 | 1816 | 14.6 |
|  | PD-L1 | 47 | 432 | 10.9 | 35 | 1391 | 2.5 | 283 | 2270 | 12.5 |
| ICI + MTT | Subtotal | N/A | N/A | N/A | 205 | 2349 | 8.7 | 142 | 986 | 14.4 |
|  | PD-1 | N/A | N/A | N/A | 52 | 1025 | 5.1 | 72 | 587 | 12.3 |
|  | PD-L1 | N/A | N/A | N/A | 153 | 1324 | 11.6 | 70 | 399 | 17.5 |
| ICI + MTT + Chemo | Subtotal | 15 | 73 | 20.5 | N/A | N/A | N/A | N/A | N/A | N/A |
|  | PD-1 | N/A | N/A | N/A | N/A | N/A | N/A | N/A | N/A | N/A |
|  | PD-L1 | 15 | 73 | 20.5 | N/A | N/A | N/A | N/A | N/A | N/A |
| Dual ICI + Chemo | Subtotal | N/A | N/A | N/A | 2 | 358 | 0.6 | 1 | 268 | 0.4 |
|  | PD-1 + CTLA-4 | N/A | N/A | N/A | 2 | 358 | 0.6 | N/A | N/A | N/A |
|  | PD-L1 + CTLA-4 | N/A | N/A | N/A | N/A | N/A | N/A | 1 | 268 | 0.4 |
| ICI + Vaccine |  | N/A | N/A | N/A | 6 | 345 | 1.7 | N/A | N/A | N/A |
| Non-ICI Containing therapy |  | N/A | N/A | N/A | 340 | 5482 | 6.20 | 421 | 3375 | 12.47 |

Abbreviations: [AE: adverse event, Chemo: chemotherapy, CTLA-4: cytotoxic T-lymphocyte antigen-4, ICI: immune checkpoint inhibitor, irAE: immune-related adverse event, Mono: Monotherapy, MTT: molecular targeted therapy, PD-1: programmed death protein-1, PD-L1: programmed death-ligand-1, TRAE: treatment related adverse event]

**Table S6.A** Incidence of Grade 3-5 Edema – Total

| **AE** | **Therapy** | **Total** | | |
| --- | --- | --- | --- | --- |
|  |  | N | n (events) | Incidence |
| **TRAE**   **N = 12,962** | ICI (Mono) | 6969 | 13 | 0.19 |
|  | ICI (Dual) | 684 | 1 | 0.15 |
|  | ICI + Chemo | 2257 | 4 | 0.18 |
|  | ICI + MTT | 2349 | 4 | 0.17 |
|  | ICI + MTT + Chemo | 0 | 0 | - |
|  | Dual ICI + Chemo | 358 | 0 | 0 |
|  | ICI + Vaccine | 345 | 2 | 0.58 |
|  | Total | 12962 | 23 | 0.18 |
| **Any Cause**   **N = 9123** | ICI (Mono) | 3354 | 3 | 0.09 |
|  | ICI (Dual) | 429 | 3 | 0.70 |
|  | ICI + Chemo | 4086 | 10 | 0.24 |
|  | ICI + MTT | 986 | 4 | 0.41 |
|  | ICI + MTT + Chemo | 0 | 0 | - |
|  | Dual ICI + Chemo | 268 | 1 | 0.37 |
|  | ICI + Vaccine | 0 | 0 | - |
|  | Total | 9123 | 21 | 0.23 |
| **irAE**   **N = 505** | ICI (Mono) | 0 | 0 | 0 |
|  | ICI (Dual) | 0 | 0 | 0 |
|  | ICI + Chemo | 432 | 0 | 0 |
|  | ICI + MTT | 0 | 0 | 0 |
|  | ICI + MTT + Chemo | 73 | 0 | 0 |
|  | Dual ICI + Chemo | 0 | 0 | 0 |
|  | ICI + Vaccine | 0 | 0 | 0 |
|  | Total | 505 | 0 | 0 |

Abbreviations [AE: adverse event, Chemo: chemotherapy, ICI: immune checkpoint inhibitor, irAE: immune-related adverse event, Mono: Monotherapy, MTT: molecular targeted therapy, TRAE: treatment related adverse event]

**Table S6.B** Incidence of Grade 3-5 Edema – By ICI Type

| **AE** | **Treatment** | **PD-1** | | | **PD-L1** | | | **CTLA-4** | | | **PD-1 + CTLA4** | | | **PD-L1 + CTLA4** | | |
| --- | --- | --- | --- | --- | --- | --- | --- | --- | --- | --- | --- | --- | --- | --- | --- | --- |
|  |  | n | N | % | n | N | % | n | N | % | n | N | % | n | N | % |
| **TRAE**   **N = 12,962** | ICI (Mono) | 6 | 4694 | 0.13 | 1 | 1557 | 0.06 | 6 | 718 | 0.84 | 0 | 0 | - | 0 | 0 | - |
|  | ICI (Dual) | 0 | 0 | - | 0 | 0 | - | 0 | 0 | - | 0 | 313 | 0 | 0 | 371 | 0 |
|  | ICI + Chemo | 2 | 866 | 0.23 | 2 | 1391 | 0.14 | 0 | 0 | - | 0 | 0 | - | 0 | 0 | - |
|  | ICI + MTT | 2 | 1025 | 0.2 | 2 | 1324 | 0.15 | 0 | 0 | - | 0 | 0 | - | 0 | 0 | - |
|  | ICI + MTT + Chemo | 0 | 0 | - | 0 | 0 | - | 0 | 0 | - | 0 | 0 | - | 0 | 0 | - |
|  | Dual ICI + Chemo | 0 | 0 | - | 0 | 0 | - | 0 | 0 | - | 0 | 358 | 0 | 0 | 0 | - |
|  | ICI + Vaccine | 2 | 345 | 0.58 | 0 | 0 | - | 0 | 0 | - | 0 | 0 | - | 0 | 0 | - |
| **Any Cause**   **N = 9123** | ICI (Mono) | 3 | 1946 | 0.15 | 0 | 1092 | 0 | 0 | 316 | 0 | 0 | 0 | - | 0 | 0 | - |
|  | ICI (Dual) | 0 | 0 | - | 0 | 0 | - | 0 | 0 | - | 2 | 256 | 0.78 | 1 | 173 | 0.58 |
|  | ICI + Chemo | 3 | 1816 | 0.17 | 7 | 2270 | 0.31 | 0 | 0 | - | 0 | 0 | - | 0 | 0 | - |
|  | ICI + MTT | 1 | 587 | 0.17 | 3 | 399 | 0.75 | 0 | 0 | - | 0 | 0 | - | 0 | 0 | - |
|  | ICI + MTT + Chemo | 0 | 0 | - | 0 | 0 | - | 0 | 0 | - | 0 | 0 | - | 0 | 0 | - |
|  | Dual ICI + Chemo | 0 | 0 | - | 0 | 0 | - | 0 | 0 | - | 0 | 0 | - | 1 | 268 | 0.37 |
|  | ICI + Vaccine | 0 | 0 | - | 0 | 0 | - | 0 | 0 | - | 0 | 0 | - | 0 | 0 | - |
| **irAE**   **N = 505** | ICI (Mono) | 0 | 0 | - | 0 | 0 | - | 0 | 0 | - | 0 | 0 | - | 0 | 0 | - |
|  | ICI (Dual) | 0 | 0 | - | 0 | 0 | - | 0 | 0 | - | 0 | 0 | - | 0 | 0 | - |
|  | ICI + Chemo | 0 | 0 | - | 0 | 432 | 0 | 0 | 0 | - | 0 | 0 | - | 0 | 0 | - |
|  | ICI + MTT | 0 | 0 | - | 0 | 0 | - | 0 | 0 | - | 0 | 0 | - | 0 | 0 | - |
|  | ICI + MTT + Chemo | 0 | 0 | - | 0 | 73 | 0 | 0 | 0 | - | 0 | 0 | - | 0 | 0 | - |
|  | Dual ICI + Chemo | 0 | 0 | - | 0 | 0 | - | 0 | 0 | - | 0 | 0 | - | 0 | 0 | - |
|  | ICI + Vaccine | 0 | 0 | - | 0 | 0 | - | 0 | 0 | - | 0 | 0 | - | 0 | 0 | - |

Abbreviations: [AE: adverse event, Chemo: chemotherapy, CTLA-4: cytotoxic T-lymphocyte antigen-4, ICI: immune checkpoint inhibitor, irAE: immune-related adverse event, Mono: Monotherapy, MTT: molecular targeted therapy, PD-1: programmed death protein-1, PD-L1: programmed death-ligand-1, TRAE: treatment related adverse event]

**Table S7 –** Meta-regression Analysis of Treatment Interruption by Grade 1-5 Any Cause/TRAE Edema and Follow Up Time

| **Outcome - Interruption** | **Type of analysis** | **Study arm N** | **Edema coeff. (95% CI)** | **Edema p** | **Follow-up coeff. (95% CI)** | **Follow-up p** | **Overall p** | **R²** | **Residual *I*²** |
| --- | --- | --- | --- | --- | --- | --- | --- | --- | --- |
| Any edema ×  AE-related | Meta-regression | 6 | 1.24 [-2.61,5.09] | 0.5269 | – | – | 0.5269 | 0 | 0.9934 |
| Any edema ×  AE-related | Adjusted by follow-up | 6 | 0.92 [-3.70,5.53] | 0.6974 | 0.24 [-0.92,1.39] | 0.6891 | 0.7885 | 0 | 0.9941 |
| TRAE edema × treatment-related | Meta-regression | 5 | -0.26 [-3.41,2.89] | 0.8697 | – | – | 0.8697 | 0 | 0.9923 |
| TRAE edema × treatment-related | Adjusted by follow-up | 5 | -0.51 [-3.08,2.06] | 0.6975 | 1.00 [-0.22,2.22] | 0.1095 | 0.2724 | 0.133 | 0.9875 |

**Figure S1.** Risk of bias assessment: (A) Risk of Bias Summary and (B) Risk of Bias Graph

A) Risk of Bias Summary


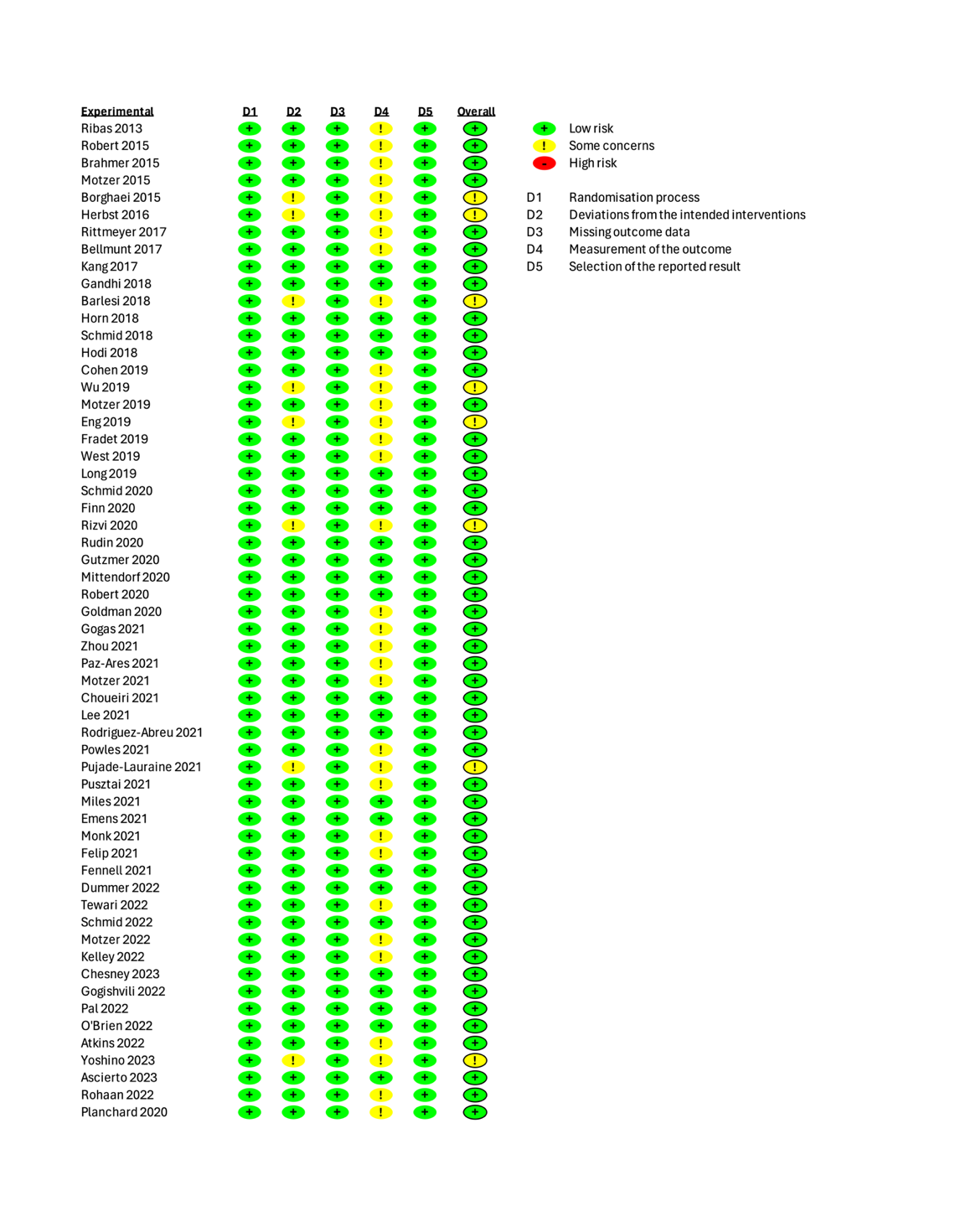


B) Risk of Bias Graph


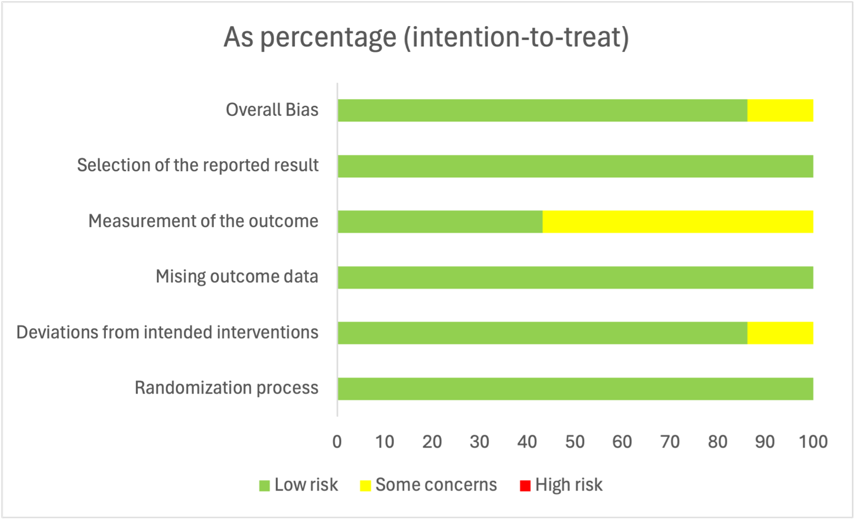


**Figure S2.** ICI monotherapy vs chemotherapy for TRAE peripheral edema. Abbreviations: [AE: Adverse event, Chemo: chemotherapy, CTLA-4: cytotoxic T-lymphocyte antigen-4, ICI: immune checkpoint inhibitor, irAE: immune-related adverse event, Mono: Monotherapy, MTT: molecular targeted therapy, OR: odds ratio, PD-1: programmed death protein-1, PD-L1: programmed death-ligand-1, TRAE: treatment related adverse event]

**Figure S2-A.** Pooled OR in patients treated with ICI monotherapy or chemotherapy. OR TRAE peripheral edema any grade.


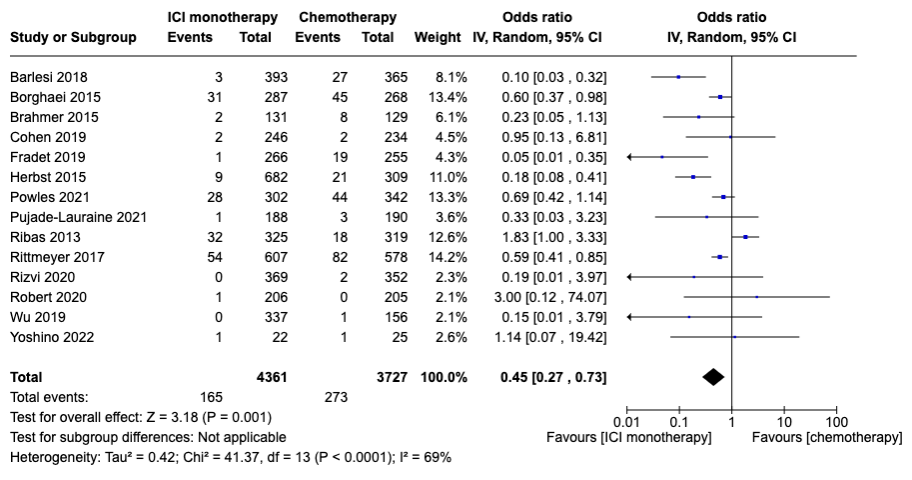


**Figure S2-B.** Pooled OR in patients treated with ICI monotherapy or chemotherapy – Subgroup analysis by ICI type. OR TRAE peripheral edema any grade.


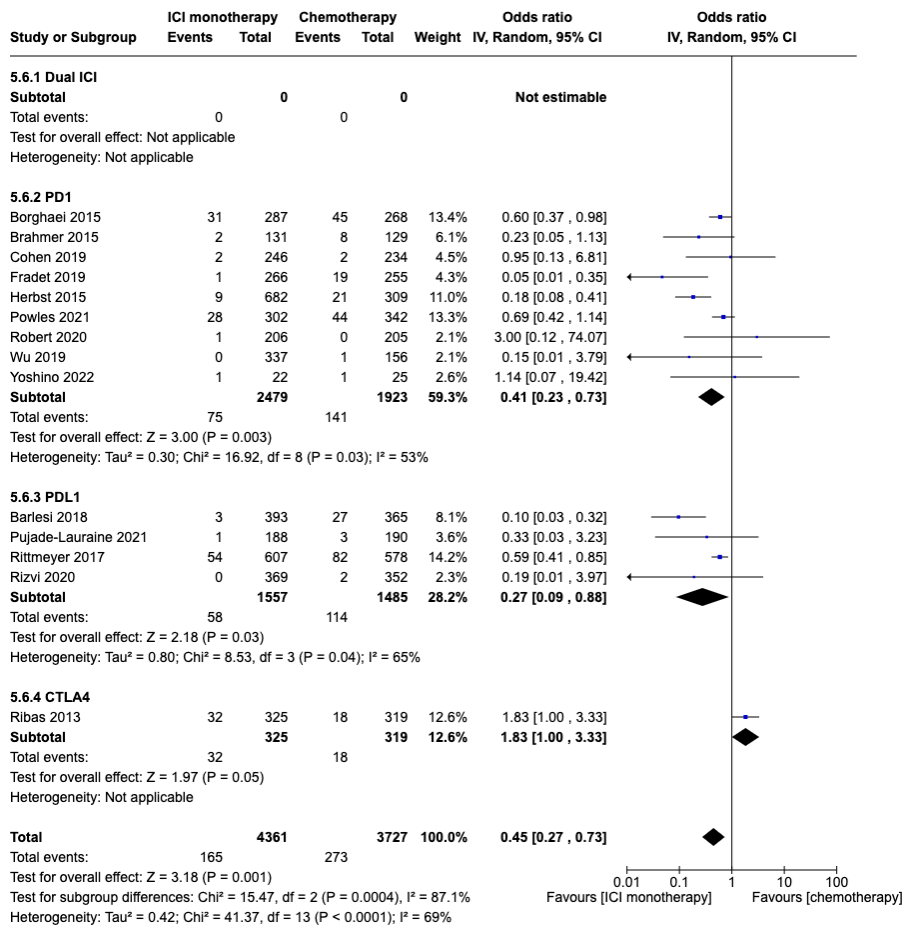


**Figure S3.** ICI monotherapy vs chemotherapy for ***Any-cause*** peripheral edema***.*** Abbreviations: [AE: Adverse event, Chemo: chemotherapy, CTLA-4: cytotoxic T-lymphocyte antigen-4, ICI: immune checkpoint inhibitor, irAE: immune-related adverse event, Mono: Monotherapy, MTT: molecular targeted therapy, OR: odds ratio, PD-1: programmed death protein-1, PD-L1: programmed death-ligand-1, TRAE: treatment related adverse event]

**Figure S3-A.** Pooled OR in patients treated with ICI monotherapy or chemotherapy. OR Any-cause peripheral edema any grade.


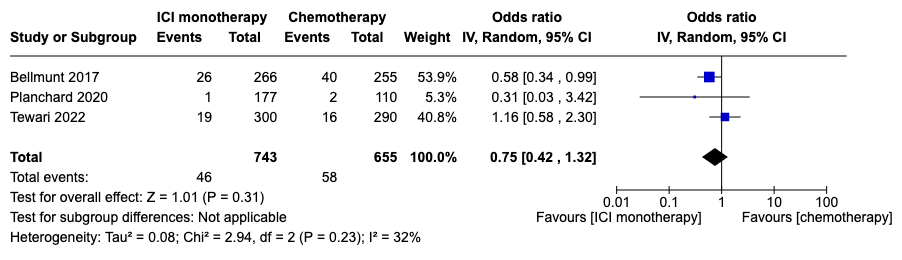


**Figure S3-B.** ICI monotherapy vs chemotherapy for ***Any-cause*** peripheral edema***.*** Pooled OR in patients treated with ICI monotherapy or chemotherapy – subgroup analysis by ICI type. OR Any-cause peripheral edema any grade.


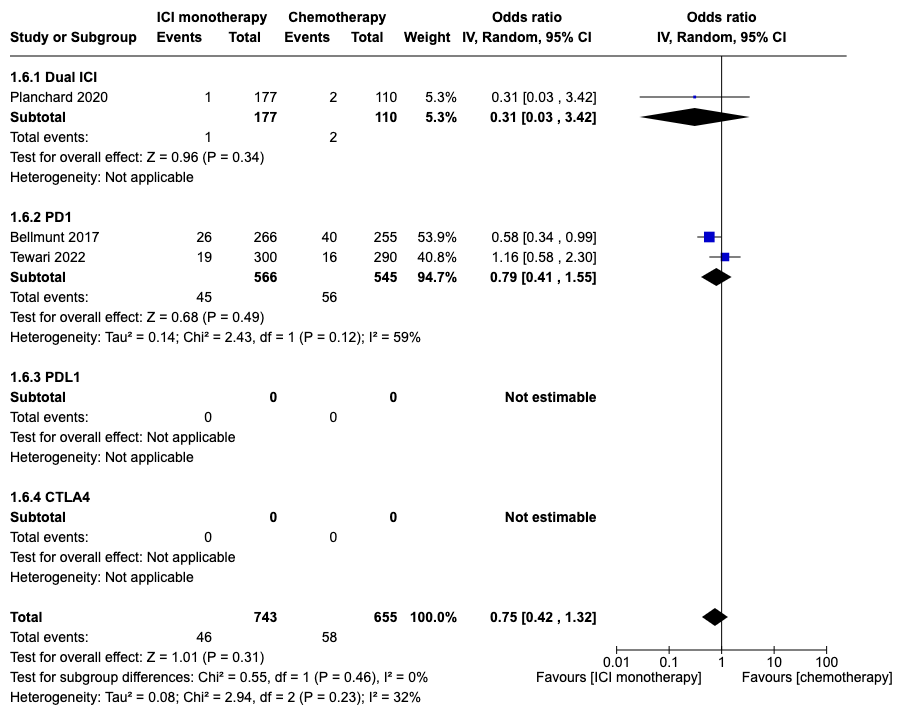


**Figure S3-C.** ICI monotherapy vs chemotherapy for ***Any-cause*** peripheral edema***.*** Pooled OR in patients treated with ICI monotherapy or chemotherapy – subgroup analysis by for non-taxane-based and taxane-based (docetaxel, paclitaxel) regimen. OR Any-cause peripheral edema any grade.


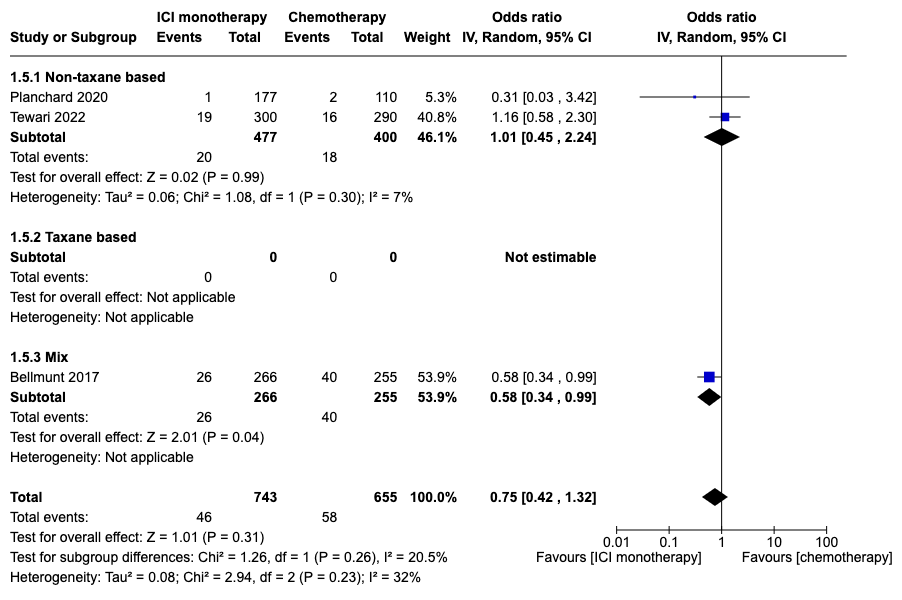


**Figure S4.** ICI + X vs X for ***TRAE*** peripheral edema. Abbreviations: [AE: Adverse event, Chemo: chemotherapy, CTLA-4: cytotoxic T-lymphocyte antigen-4, ICI: immune checkpoint inhibitor, irAE: immune-related adverse event, Mono: Monotherapy, MTT: molecular targeted therapy, OR: odds ratio, PD-1: programmed death protein-1, PD-L1: programmed death-ligand-1, TRAE: treatment related adverse event]

**Figure S4-A.** Pooled OR in patients treated with the addition of ICI or control. OR ***TRAE*** peripheral edema any grade.


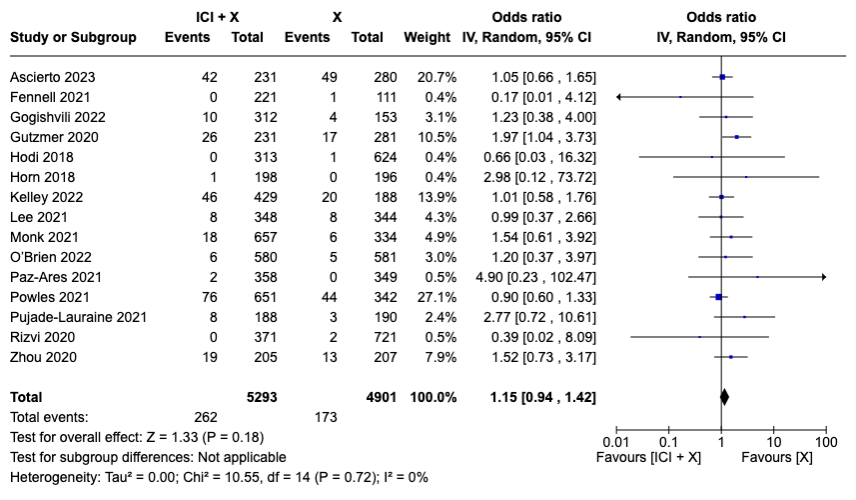


**Figure S4-B.** Pooled OR in patients treated with the addition of ICI or control- Subgroup analysis for ICI types. OR ***TRAE*** peripheral edema any grade.


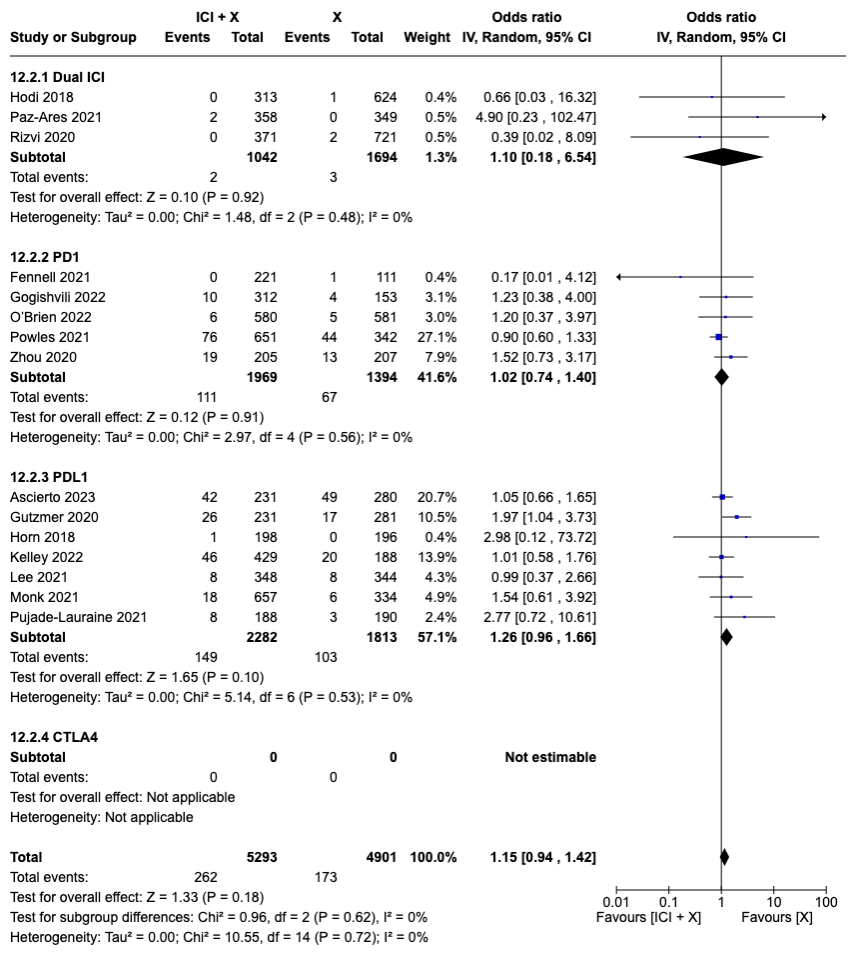


**Figure S4-C.** Pooled OR in patients treated with the addition of ICI or control- Subgroup analysis by control type. OR ***TRAE*** peripheral edema any grade.


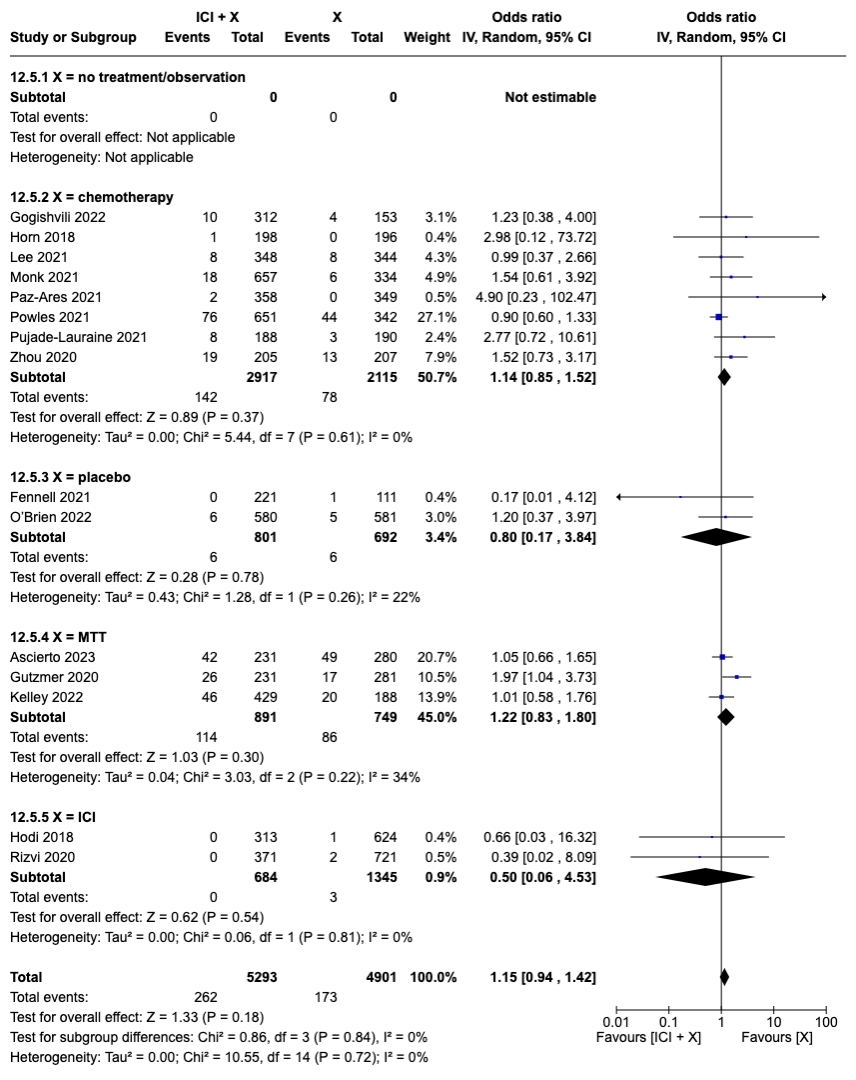


**Figure S5.** ICI + chemotherapy vs chemotherapy for TRAE peripheral edema. Abbreviations: [AE: Adverse event, Chemo: chemotherapy, CTLA-4: cytotoxic T-lymphocyte antigen-4, ICI: immune checkpoint inhibitor, irAE: immune-related adverse event, Mono: Monotherapy, MTT: molecular targeted therapy, OR: odds ratio, PD-1: programmed death protein-1, PD-L1: programmed death-ligand-1, TRAE: treatment related adverse event]

**Figure S5-A.** Pooled OR in patients treated with ICI with chemotherapy or chemotherapy. OR TRAE peripheral edema any grade


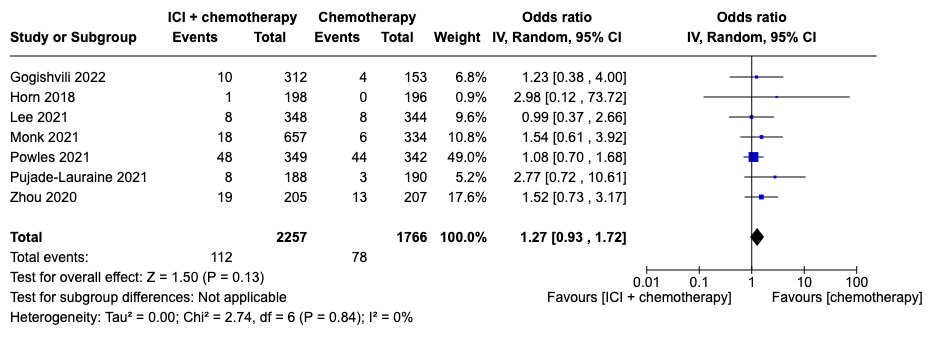


**Figure S5-B.** Pooled OR in patients treated with ICI with chemotherapy or chemotherapy- Subgroup analysis for non-taxane-based and taxane-based (docetaxel, paclitaxel) regimen any grade OR TRAE peripheral edema any grade


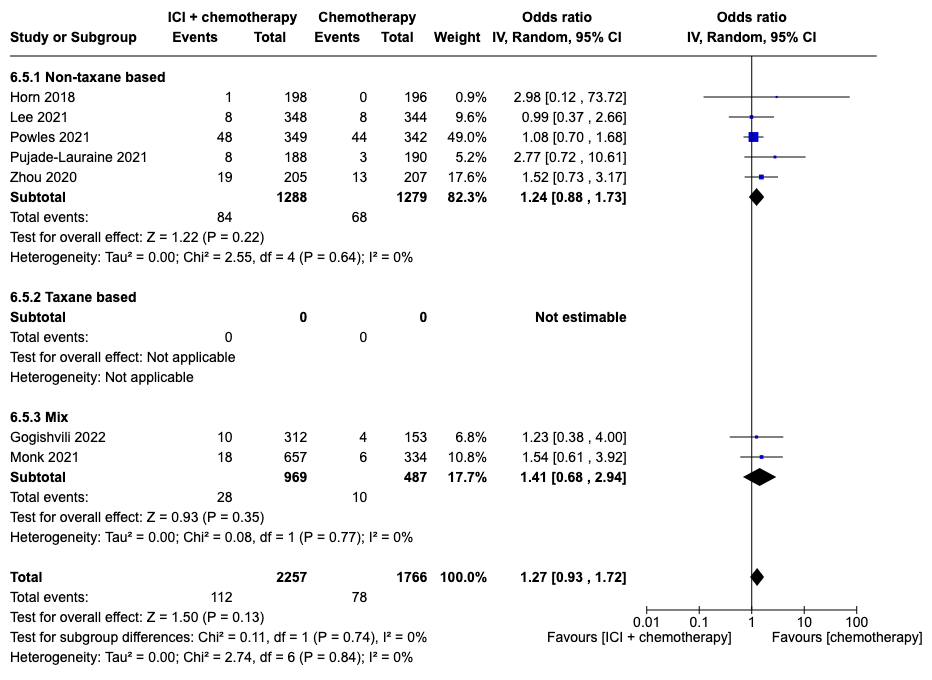


**Figure S5-C.** Pooled OR in patients treated with ICI with chemotherapy or chemotherapy- Subgroup analysis for ICI subtypes any grade. OR TRAE peripheral edema any grade


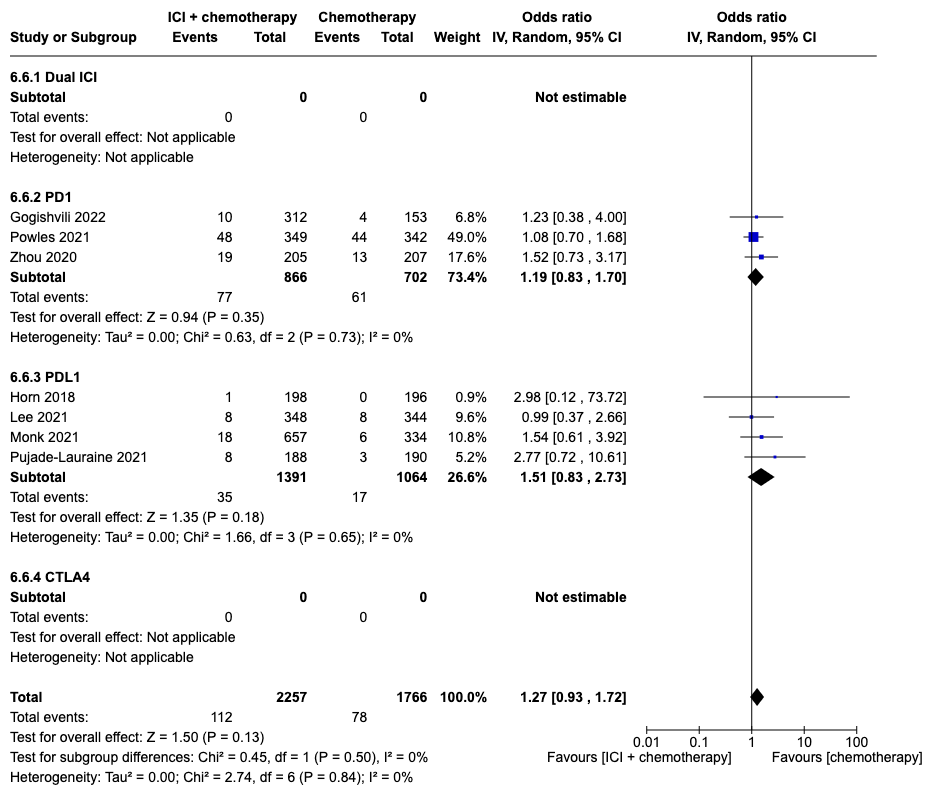


**Figure S6.** ICI + MTT vs MTT for TRAE peripheral edema. Abbreviations: [AE: Adverse event, Chemo: chemotherapy, CTLA-4: cytotoxic T-lymphocyte antigen-4, ICI: immune checkpoint inhibitor, irAE: immune-related adverse event, Mono: Monotherapy, MTT: molecular targeted therapy, OR: odds ratio, PD-1: programmed death protein-1, PD-L1: programmed death-ligand-1, TRAE: treatment related adverse event]

**Figure S6-A.** Pooled OR in patients treated with ICI with MTT or MTT. 6 studies were included. OR TRAE peripheral edema any grade


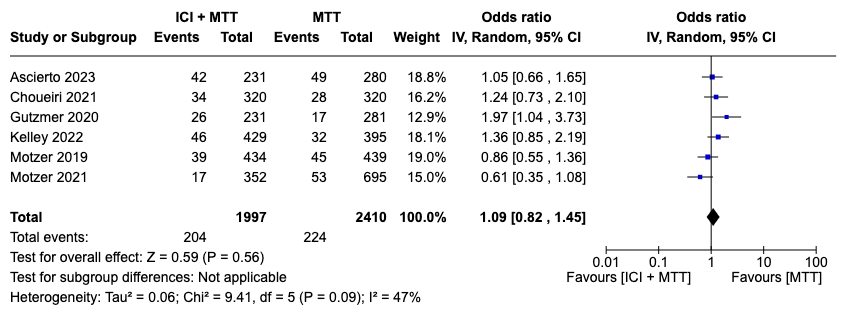


**Figure S6-B.** Pooled OR in patients treated with ICI with MTT or MTT- Subgroup analysis for ICI subtypes any grade. OR TRAE peripheral edema any grade


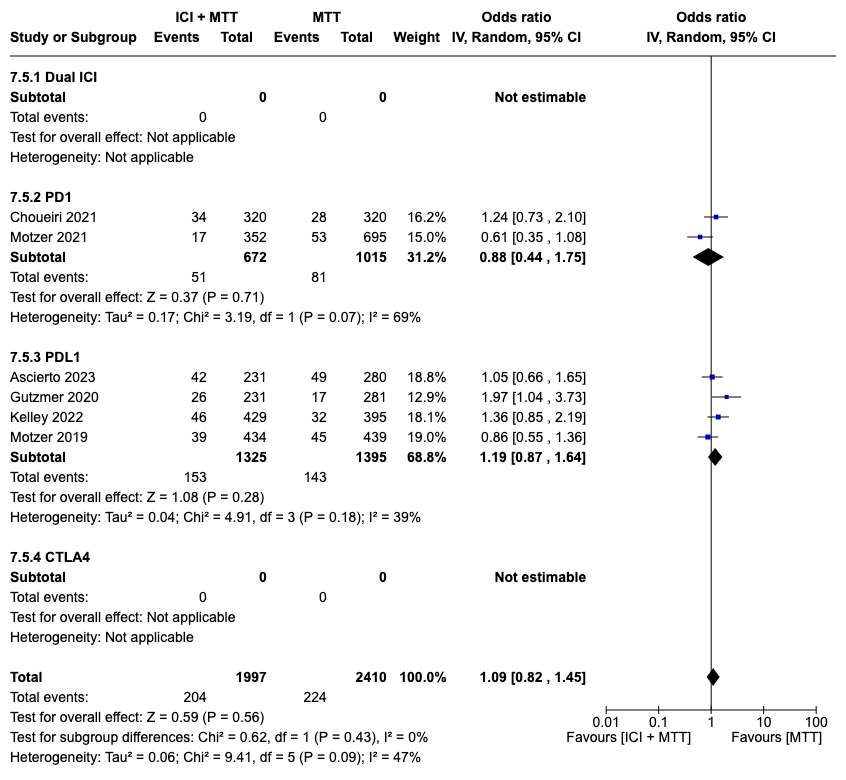


**Figure S7.** ICI + X vs X for ***Any-cause*** peripheral edema. Abbreviations: [AE: Adverse event, Chemo: chemotherapy, CTLA-4: cytotoxic T-lymphocyte antigen-4, ICI: immune checkpoint inhibitor, irAE: immune-related adverse event, Mono: Monotherapy, MTT: molecular targeted therapy, OR: odds ratio, PD-1: programmed death protein-1, PD-L1: programmed death-ligand-1, TRAE: treatment related adverse event]

**Figure S7-A.** Pooled OR in patients treated with the addition of ICI or control. OR ***Any-cause*** peripheral edema any grade.


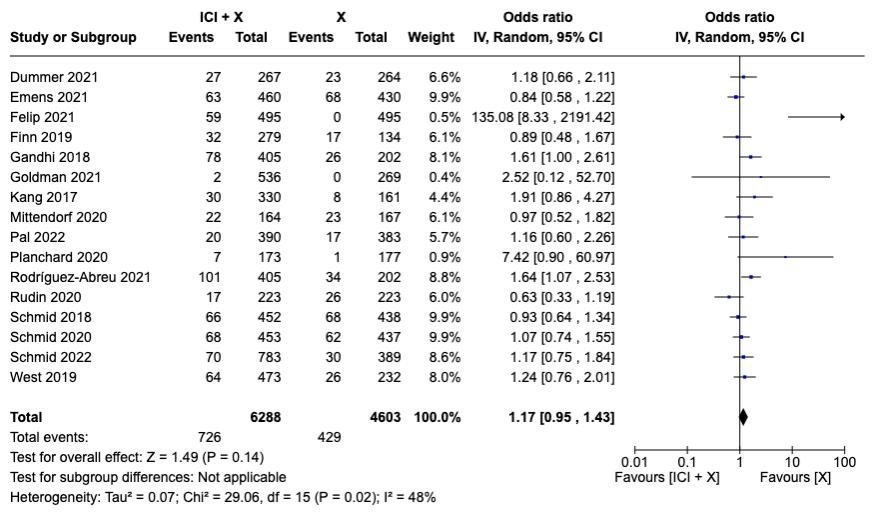


**Figure S7-B.** Pooled OR in patients treated with the addition of ICI or control - subgroup analysis for ICI subtypes. OR ***Any-cause*** peripheral edema any grade


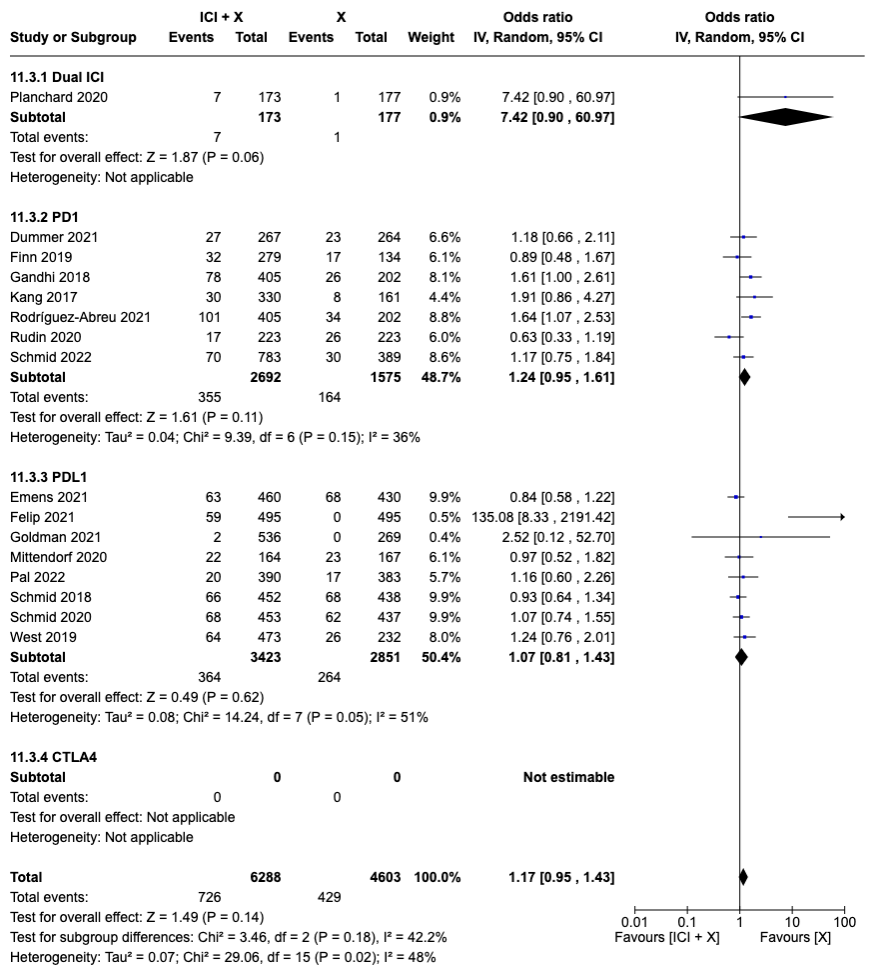


**Figure S8.** ICI + chemotherapy vs chemotherapy for ***Any-cause*** peripheral edema. Abbreviations: [AE: Adverse event, Chemo: chemotherapy, CTLA-4: cytotoxic T-lymphocyte antigen-4, ICI: immune checkpoint inhibitor, irAE: immune-related adverse event, Mono: Monotherapy, MTT: molecular targeted therapy, OR: odds ratio, PD-1: programmed death protein-1, PD-L1: programmed death-ligand-1, TRAE: treatment related adverse event]

**Figure S8-A.**  Pooled OR in patients treated with ICI with chemotherapy or chemotherapy. OR Any-cause peripheral edema any grade


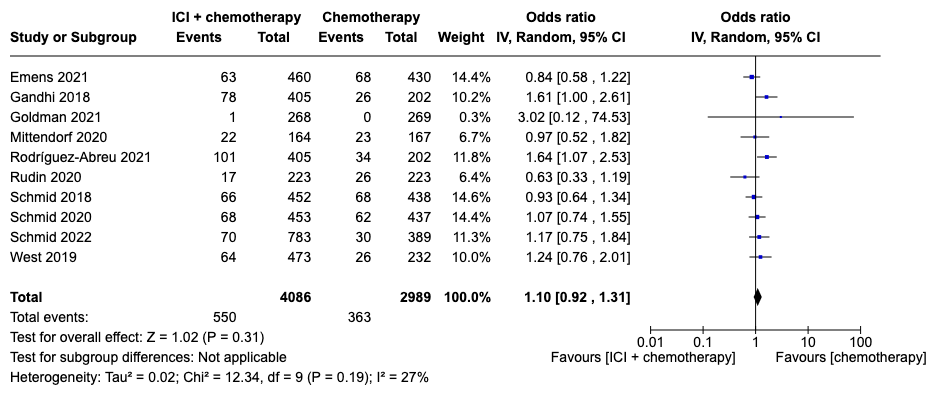


**Figure S8-B.** Pooled OR in patients treated with ICI with chemotherapy or chemotherapy - Subgroup analysis for non-taxane-based and taxane-based (docetaxel, paclitaxel) regimen. OR ***Any-cause*** peripheral edema any grade


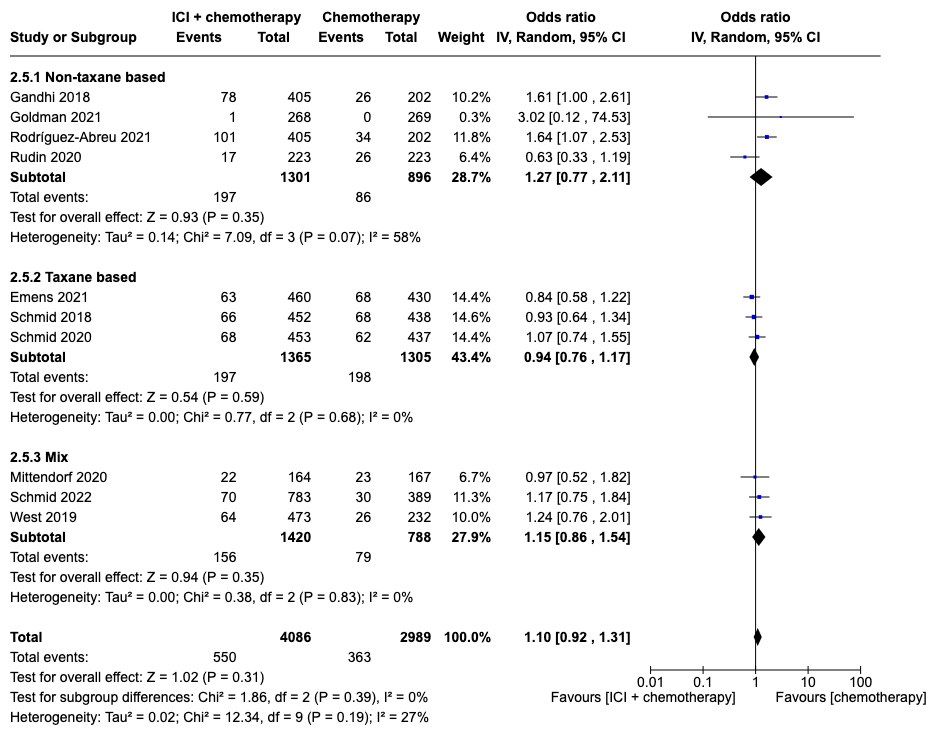


**Figure S8- C.** Pooled OR in patients treated with ICI with chemotherapy or chemotherapy - Subgroup analysis for ICI subtypes any grade. OR ***Any-cause*** peripheral edema any grade


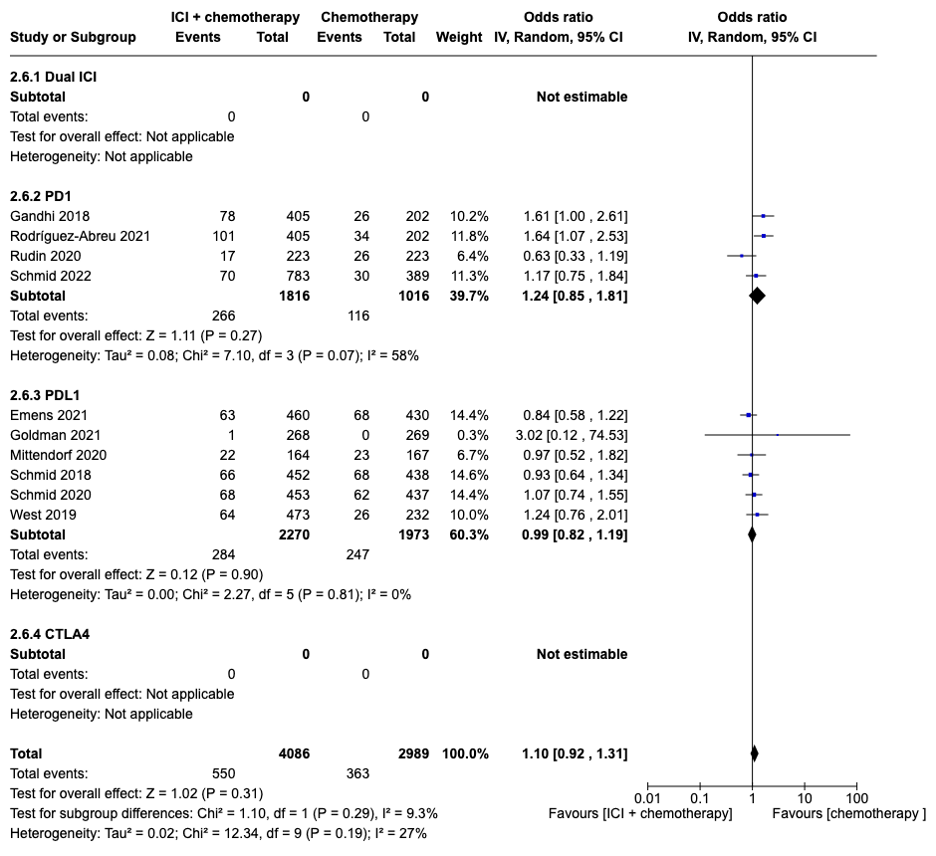


**Figure S9.** ICI + MTT vs MTT for ***Any-cause*** peripheral edema. Abbreviations: [AE: Adverse event, Chemo: chemotherapy, CTLA-4: cytotoxic T-lymphocyte antigen-4, ICI: immune checkpoint inhibitor, irAE: immune-related adverse event, Mono: Monotherapy, MTT: molecular targeted therapy, OR: odds ratio, PD-1: programmed death protein-1, PD-L1: programmed death-ligand-1, TRAE: treatment related adverse event]

**Figure S9-A**. Pooled OR in patients treated with ICI with MTT or MTT. OR ***Any-cause*** peripheral edema any grade


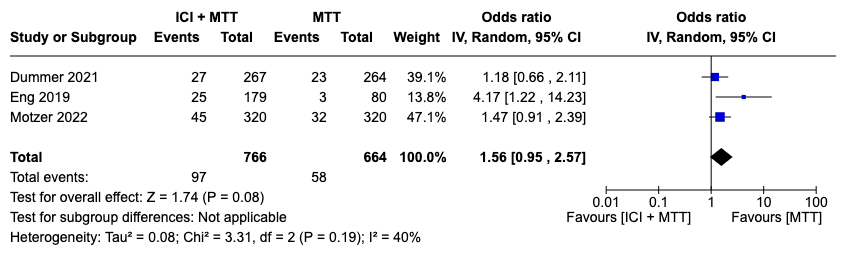


**Figure S9-B**. Pooled OR in patients treated with ICI with MTT or MTT – Subgroup analysis by ICI type. OR ***Any-cause*** peripheral edema any grade


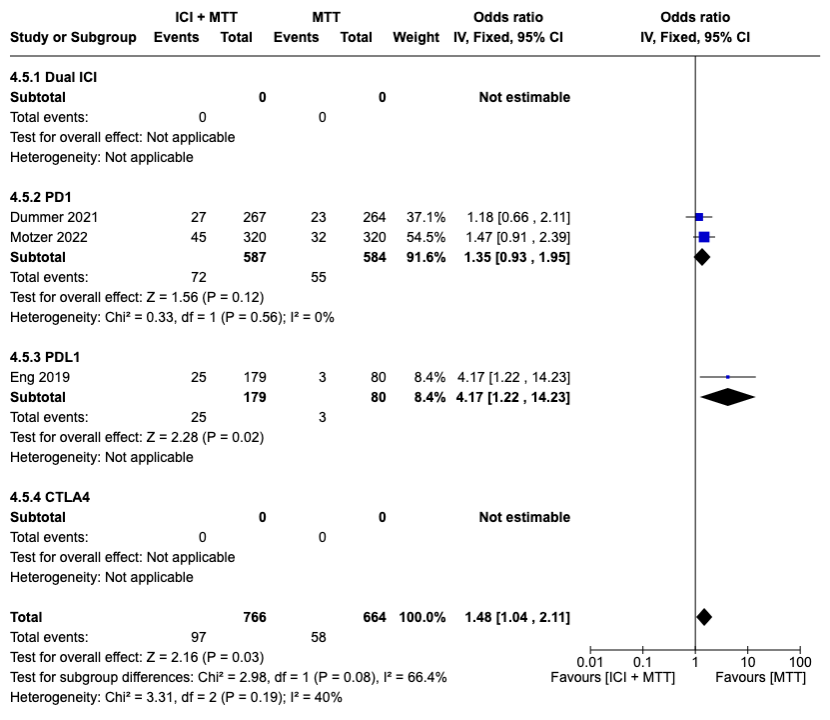


**Figure S10-** Flowchart of the systematic review process for management/treatment outcomes of irAE peripheral edema. 342 articles were removed due to not being relevant to the study question, and 115 studies underwent further screening. 34 were removed as they either had wrong outcomes (24), wrong intervention (1) , wrong study design (2), wrong setting (5), or wrong language (2) during this screening process. Of those 81 studies, 19 articles (17 case reports/series, 1 phase 1/2 clinical trial, 1 pharmacovigilance study) specifically reported on peripheral edema and were selected for use).


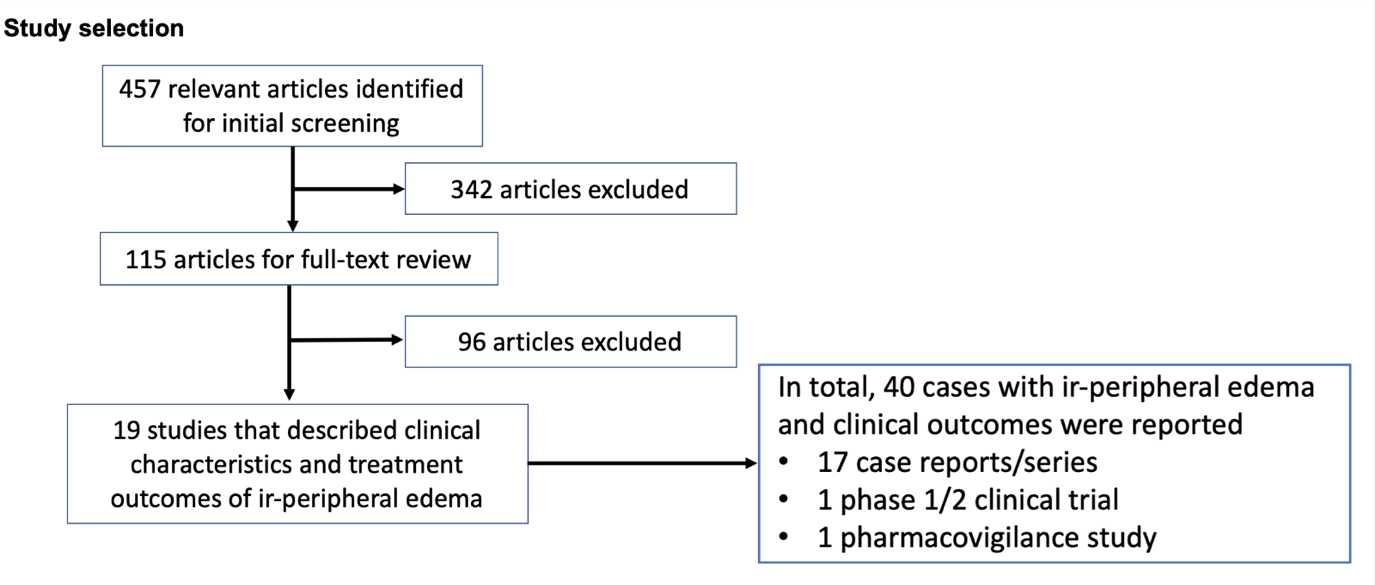


**Reference S1 - Supplementary Works Cited**

1. Felip E, Altorki N, Zhou C, et al. Adjuvant atezolizumab after adjuvant chemotherapy in resected stage IB-IIIA non-small-cell lung cancer (IMpower010): a randomised, multicentre, open-label, phase 3 trial. Lancet. 2021;398(10308):1344-1357. doi:10.1016/S0140-6736(21)02098-5

2. Pal SK, Uzzo R, Karam JA, et al. Adjuvant atezolizumab versus placebo for patients with renal cell carcinoma at increased risk of recurrence following resection (IMmotion010): a multicentre, randomised, double-blind, phase 3 trial. Lancet. 2022;400(10358):1103-1116. doi:10.1016/S0140-6736(22)01658-0

3. Planchard D, Reinmuth N, Orlov S, et al. ARCTIC: durvalumab with or without tremelimumab as third-line or later treatment of metastatic non-small-cell lung cancer. Ann Oncol. 2020;31(5):609-618. doi:10.1016/j.annonc.2020.02.006

4. Schmid P, Adams S, Rugo HS, et al. Atezolizumab and Nab-Paclitaxel in Advanced Triple-Negative Breast Cancer. N Engl J Med. 2018;379(22):2108-2121. doi:10.1056/NEJMoa1809615

5. West H, McCleod M, Hussein M, et al. Atezolizumab in combination with carboplatin plus nab-paclitaxel chemotherapy compared with chemotherapy alone as first-line treatment for metastatic non-squamous non-small-cell lung cancer (IMpower130): a multicentre, randomised, open-label, phase 3 trial. Lancet Oncol. 2019;20(7):924-937. doi:10.1016/S1470-2045(19)30167-6

6. Schmid P, Rugo HS, Adams S, et al. Atezolizumab plus nab-paclitaxel as first-line treatment for unresectable, locally advanced or metastatic triple-negative breast cancer (IMpassion130): updated efficacy results from a randomised, double-blind, placebo-controlled, phase 3 trial. Lancet Oncol. 2020;21(1):44-59. doi:10.1016/S1470-2045(19)30689-8

7. Rittmeyer A, Barlesi F, Waterkamp D, et al. Atezolizumab versus docetaxel in patients with previously treated non-small-cell lung cancer (OAK): a phase 3, open-label, multicentre randomised controlled trial. Lancet. 2017;389(10066):255-265. doi:10.1016/S0140-6736(16)32517-X

8. Eng C, Kim TW, Bendell J, et al. Atezolizumab with or without cobimetinib versus regorafenib in previously treated metastatic colorectal cancer (IMblaze370): a multicentre, open-label, phase 3, randomised, controlled trial. Lancet Oncol. 2019;20(6):849-861. doi:10.1016/S1470-2045(19)30027-0

9. Gutzmer R, Stroyakovskiy D, Gogas H, et al. Atezolizumab, vemurafenib, and cobimetinib as first-line treatment for unresectable advanced BRAFV600 mutation-positive melanoma (IMspire150): primary analysis of the randomised, double-blind, placebo-controlled, phase 3 trial. Lancet. 2020;395(10240):1835-1844. doi:10.1016/S0140-6736(20)30934-X

10. Pujade-Lauraine E, Fujiwara K, Ledermann JA, et al. Avelumab alone or in combination with chemotherapy versus chemotherapy alone in platinum-resistant or platinum-refractory ovarian cancer (JAVELIN Ovarian 200): an open-label, three-arm, randomised, phase 3 study. Lancet Oncol. 2021;22(7):1034-1046. doi:10.1016/S1470-2045(21)00216-3

11. Motzer RJ, Penkov K, Haanen J, et al. Avelumab plus Axitinib versus Sunitinib for Advanced Renal-Cell Carcinoma. N Engl J Med. 2019;380(12):1103-1115. doi:10.1056/NEJMoa1816047

12. Lee NY, Ferris RL, Psyrri A, et al. Avelumab plus standard-of-care chemoradiotherapy versus chemoradiotherapy alone in patients with locally advanced squamous cell carcinoma of the head and neck: a randomised, double-blind, placebo-controlled, multicentre, phase 3 trial. Lancet Oncol. 2021;22(4):450-462. doi:10.1016/S1470-2045(20)30737-3

13. Barlesi F, Vansteenkiste J, Spigel D, et al. Avelumab versus docetaxel in patients with platinum-treated advanced non-small-cell lung cancer (JAVELIN Lung 200): an open-label, randomised, phase 3 study. Lancet Oncol. 2018;19(11):1468-1479. doi:10.1016/S1470-2045(18)30673-9

14. Kelley RK, Rimassa L, Cheng AL, et al. Cabozantinib plus atezolizumab versus sorafenib for advanced hepatocellular carcinoma (COSMIC-312): a multicentre, open-label, randomised, phase 3 trial. Lancet Oncol. 2022;23(8):995-1008. doi:10.1016/S1470-2045(22)00326-6

15. Zhou C, Chen G, Huang Y, et al. Camrelizumab plus carboplatin and pemetrexed versus chemotherapy alone in chemotherapy-naive patients with advanced non-squamous non-small-cell lung cancer (CameL): a randomised, open-label, multicentre, phase 3 trial. Lancet Respir Med. 2021;9(3):305-314. doi:10.1016/S2213-2600(20)30365-9

16. Gogishvili M, Melkadze T, Makharadze T, et al. Cemiplimab plus chemotherapy versus chemotherapy alone in non-small cell lung cancer: a randomized, controlled, double-blind phase 3 trial. Nat Med. 2022;28(11):2374-2380. doi:10.1038/s41591-022-01977-y

17. Monk BJ, Colombo N, Oza AM, et al. Chemotherapy with or without avelumab followed by avelumab maintenance versus chemotherapy alone in patients with previously untreated epithelial ovarian cancer (JAVELIN Ovarian 100): an open-label, randomised, phase 3 trial. Lancet Oncol. 2021;22(9):1275-1289. doi:10.1016/S1470-2045(21)00342-9

18. Gogas H, Dréno B, Larkin J, et al. Cobimetinib plus atezolizumab in BRAFV600 wild-type melanoma: primary results from the randomized phase III IMspire170 study. Ann Oncol. 2021;32(3):384-394. doi:10.1016/j.annonc.2020.12.004

19. Atkins MB, Lee SJ, Chmielowski B, et al. Combination Dabrafenib and Trametinib Versus Combination Nivolumab and Ipilimumab for Patients With Advanced BRAF-Mutant Melanoma: The DREAMseq Trial-ECOG-ACRIN EA6134. J Clin Oncol. 2023;41(2):186-197. doi:10.1200/JCO.22.01763

20. Pusztai L, Yau C, Wolf DM, et al. Durvalumab with olaparib and paclitaxel for high-risk HER2-negative stage II/III breast cancer: Results from the adaptively randomized I-SPY2 trial. Cancer Cell. 2021;39(7):989-998.e5. doi:10.1016/j.ccell.2021.05.009

21. Rizvi NA, Cho BC, Reinmuth N, et al. Durvalumab With or Without Tremelimumab vs Standard Chemotherapy in First-line Treatment of Metastatic Non-Small Cell Lung Cancer: The MYSTIC Phase 3 Randomized Clinical Trial. JAMA Oncol. 2020;6(5):661-674. doi:10.1001/jamaoncol.2020.0237

22. Goldman JW, Dvorkin M, Chen Y, et al. Durvalumab, with or without tremelimumab, plus platinum-etoposide versus platinum-etoposide alone in first-line treatment of extensive-stage small-cell lung cancer (CASPIAN): updated results from a randomised, controlled, open-label, phase 3 trial. Lancet Oncol. 2021;22(1):51-65. doi:10.1016/S1470-2045(20)30539-8

23. Long GV, Dummer R, Hamid O, et al. Epacadostat plus pembrolizumab versus placebo plus pembrolizumab in patients with unresectable or metastatic melanoma (ECHO-301/KEYNOTE-252): a phase 3, randomised, double-blind study. Lancet Oncol. 2019;20(8):1083-1097. doi:10.1016/S1470-2045(19)30274-8

24. Schmid P, Cortes J, Dent R, et al. Event-free Survival with Pembrolizumab in Early Triple-Negative Breast Cancer. N Engl J Med. 2022;386(6):556-567. doi:10.1056/NEJMoa2112651

25. Horn L, Mansfield AS, Szczęsna A, et al. First-Line Atezolizumab plus Chemotherapy in Extensive-Stage Small-Cell Lung Cancer. N Engl J Med. 2018;379(23):2220-2229. doi:10.1056/NEJMoa1809064

26. Emens LA, Adams S, Barrios CH, et al. First-line atezolizumab plus nab-paclitaxel for unresectable, locally advanced, or metastatic triple-negative breast cancer: IMpassion130 final overall survival analysis. Ann Oncol. 2021;32(8):983-993. doi:10.1016/j.annonc.2021.05.355

27. Paz-Ares L, Ciuleanu TE, Cobo M, et al. First-line nivolumab plus ipilimumab combined with two cycles of chemotherapy in patients with non-small-cell lung cancer (CheckMate 9LA): an international, randomised, open-label, phase 3 trial. Lancet Oncol. 2021;22(2):198-211. doi:10.1016/S1470-2045(20)30641-0

28. Robert C, Long GV, Brady B, et al. Five-Year Outcomes With Nivolumab in Patients With Wild-Type BRAF Advanced Melanoma. J Clin Oncol. 2020;38(33):3937-3946. doi:10.1200/JCO.20.00995

29. Motzer R, Alekseev B, Rha SY, et al. Lenvatinib plus Pembrolizumab or Everolimus for Advanced Renal Cell Carcinoma. N Engl J Med. 2021;384(14):1289-1300. doi:10.1056/NEJMoa2035716

30. Mittendorf EA, Zhang H, Barrios CH, et al. Neoadjuvant atezolizumab in combination with sequential nab-paclitaxel and anthracycline-based chemotherapy versus placebo and chemotherapy in patients with early-stage triple-negative breast cancer (IMpassion031): a randomised, double-blind, phase 3 trial. Lancet. 2020;396(10257):1090-1100. doi:10.1016/S0140-6736(20)31953-X

31. Kang YK, Boku N, Satoh T, et al. Nivolumab in patients with advanced gastric or gastro-oesophageal junction cancer refractory to, or intolerant of, at least two previous chemotherapy regimens (ONO-4538-12, ATTRACTION-2): a randomised, double-blind, placebo-controlled, phase 3 trial. Lancet. 2017;390(10111):2461-2471. doi:10.1016/S0140-6736(17)31827-5

32. Choueiri TK, Powles T, Burotto M, et al. Nivolumab plus Cabozantinib versus Sunitinib for Advanced Renal-Cell Carcinoma. N Engl J Med. 2021;384(9):829-841. doi:10.1056/NEJMoa2026982

33. Motzer RJ, Powles T, Burotto M, et al. Nivolumab plus cabozantinib versus sunitinib in first-line treatment for advanced renal cell carcinoma (CheckMate 9ER): long-term follow-up results from an open-label, randomised, phase 3 trial. Lancet Oncol. 2022;23(7):888-898. doi:10.1016/S1470-2045(22)00290-X

34. Hodi FS, Chiarion-Sileni V, Gonzalez R, et al. Nivolumab plus ipilimumab or nivolumab alone versus ipilimumab alone in advanced melanoma (CheckMate 067): 4-year outcomes of a multicentre, randomised, phase 3 trial. Lancet Oncol. 2018;19(11):1480-1492. doi:10.1016/S1470-2045(18)30700-9

35. Wu YL, Lu S, Cheng Y, et al. Nivolumab Versus Docetaxel in a Predominantly Chinese Patient Population With Previously Treated Advanced NSCLC: CheckMate 078 Randomized Phase III Clinical Trial. J Thorac Oncol. 2019;14(5):867-875. doi:10.1016/j.jtho.2019.01.006

36. Borghaei H, Paz-Ares L, Horn L, et al. Nivolumab versus Docetaxel in Advanced Nonsquamous Non-Small-Cell Lung Cancer. N Engl J Med. 2015;373(17):1627-1639. doi:10.1056/NEJMoa1507643

37. Brahmer J, Reckamp KL, Baas P, et al. Nivolumab versus Docetaxel in Advanced Squamous-Cell Non-Small-Cell Lung Cancer. N Engl J Med. 2015;373(2):123-135. doi:10.1056/NEJMoa1504627

38. Motzer RJ, Escudier B, McDermott DF, et al. Nivolumab versus Everolimus in Advanced Renal-Cell Carcinoma. N Engl J Med. 2015;373(19):1803-1813. doi:10.1056/NEJMoa1510665

39. Fennell DA, Ewings S, Ottensmeier C, et al. Nivolumab versus placebo in patients with relapsed malignant mesothelioma (CONFIRM): a multicentre, double-blind, randomised, phase 3 trial. Lancet Oncol. 2021;22(11):1530-1540. doi:10.1016/S1470-2045(21)00471-X

40. Ascierto PA, Stroyakovskiy D, Gogas H, et al. Overall survival with first-line atezolizumab in combination with vemurafenib and cobimetinib in BRAFV600 mutation-positive advanced melanoma (IMspire150): second interim analysis of a multicentre, randomised, phase 3 study. Lancet Oncol. 2023;24(1):33-44. doi:10.1016/S1470-2045(22)00687-8

41. Powles T, Csőszi T, Özgüroğlu M, et al. Pembrolizumab alone or combined with chemotherapy versus chemotherapy as first-line therapy for advanced urothelial carcinoma (KEYNOTE-361): a randomised, open-label, phase 3 trial. Lancet Oncol. 2021;22(7):931-945. doi:10.1016/S1470-2045(21)00152-2

42. Bellmunt J, de Wit R, Vaughn DJ, et al. Pembrolizumab as Second-Line Therapy for Advanced Urothelial Carcinoma. N Engl J Med. 2017;376(11):1015-1026. doi:10.1056/NEJMoa1613683

43. Finn RS, Ryoo BY, Merle P, et al. Pembrolizumab As Second-Line Therapy in Patients With Advanced Hepatocellular Carcinoma in KEYNOTE-240: A Randomized, Double-Blind, Phase III Trial. J Clin Oncol. 2020;38(3):193-202. doi:10.1200/JCO.19.01307

44. Yoshino T, Andre T, Kim TW, et al. Pembrolizumab in Asian patients with microsatellite-instability-high/mismatch-repair-deficient colorectal cancer. Cancer Sci. 2023;114(3):1026-1036. doi:10.1111/cas.15650

45. Rudin CM, Awad MM, Navarro A, et al. Pembrolizumab or Placebo Plus Etoposide and Platinum as First-Line Therapy for Extensive-Stage Small-Cell Lung Cancer: Randomized, Double-Blind, Phase III KEYNOTE-604 Study. J Clin Oncol. 2020;38(21):2369-2379. doi:10.1200/JCO.20.00793

46. Gandhi L, Rodríguez-Abreu D, Gadgeel S, et al. Pembrolizumab plus Chemotherapy in Metastatic Non-Small-Cell Lung Cancer. N Engl J Med. 2018;378(22):2078-2092. doi:10.1056/NEJMoa1801005

47. Herbst RS, Baas P, Kim DW, et al. Pembrolizumab versus docetaxel for previously treated, PD-L1-positive, advanced non-small-cell lung cancer (KEYNOTE-010): a randomised controlled trial. Lancet. 2016;387(10027):1540-1550. doi:10.1016/S0140-6736(15)01281-7

48. Robert C, Schachter J, Long GV, et al. Pembrolizumab versus Ipilimumab in Advanced Melanoma. N Engl J Med. 2015;372(26):2521-2532. doi:10.1056/NEJMoa1503093

49. Cohen EEW, Soulières D, Le Tourneau C, et al. Pembrolizumab versus methotrexate, docetaxel, or cetuximab for recurrent or metastatic head-and-neck squamous cell carcinoma (KEYNOTE-040): a randomised, open-label, phase 3 study. Lancet. 2019;393(10167):156-167. doi:10.1016/S0140-6736(18)31999-8

50. O’Brien M, Paz-Ares L, Marreaud S, et al. Pembrolizumab versus placebo as adjuvant therapy for completely resected stage IB-IIIA non-small-cell lung cancer (PEARLS/KEYNOTE-091): an interim analysis of a randomised, triple-blind, phase 3 trial. Lancet Oncol. 2022;23(10):1274-1286. doi:10.1016/S1470-2045(22)00518-6

51. Rodríguez-Abreu D, Powell SF, Hochmair MJ, et al. Pemetrexed plus platinum with or without pembrolizumab in patients with previously untreated metastatic nonsquamous NSCLC: protocol-specified final analysis from KEYNOTE-189. Ann Oncol. 2021;32(7):881-895. doi:10.1016/j.annonc.2021.04.008

52. Ribas A, Kefford R, Marshall MA, et al. Phase III randomized clinical trial comparing tremelimumab with standard-of-care chemotherapy in patients with advanced melanoma. J Clin Oncol. 2013;31(5):616-622. doi:10.1200/JCO.2012.44.6112

53. Miles D, Gligorov J, André F, et al. Primary results from IMpassion131, a double-blind, placebo-controlled, randomised phase III trial of first-line paclitaxel with or without atezolizumab for unresectable locally advanced/metastatic triple-negative breast cancer. Ann Oncol. 2021;32(8):994-1004. doi:10.1016/j.annonc.2021.05.801

54. Fradet Y, Bellmunt J, Vaughn DJ, et al. Randomized phase III KEYNOTE-045 trial of pembrolizumab versus paclitaxel, docetaxel, or vinflunine in recurrent advanced urothelial cancer: results of >2 years of follow-up. Ann Oncol. 2019;30(6):970-976. doi:10.1093/annonc/mdz127

55. Dummer R, Long GV, Robert C, et al. Randomized Phase III Trial Evaluating Spartalizumab Plus Dabrafenib and Trametinib for BRAF V600-Mutant Unresectable or Metastatic Melanoma. J Clin Oncol. 2022;40(13):1428-1438. doi:10.1200/JCO.21.01601

56. Chesney JA, Ribas A, Long GV, et al. Randomized, Double-Blind, Placebo-Controlled, Global Phase III Trial of Talimogene Laherparepvec Combined With Pembrolizumab for Advanced Melanoma. J Clin Oncol. 2023;41(3):528-540. doi:10.1200/JCO.22.00343

57. Tewari KS, Monk BJ, Vergote I, et al. Survival with Cemiplimab in Recurrent Cervical Cancer. N Engl J Med. 2022;386(6):544-555. doi:10.1056/NEJMoa2112187

58. Rohaan MW, Borch TH, van den Berg JH, et al. Tumor-Infiltrating Lymphocyte Therapy or Ipilimumab in Advanced Melanoma. N Engl J Med. 2022;387(23):2113-2125. doi:10.1056/NEJMoa2210233

**References S2- Supplementary Works Cited**

1. Bas O, Ozbek A, Guven D, et al. Pembrolizumab- and/or pazopanib-induced remitting seronegative symmetrical synovitis with pitting edema in a patient with renal cell carcinoma. *Journal of Oncology Pharmacy Practice*. 2019;26(5):1230-1233. doi:10.1177/1078155219884113
2. Amrane K, Meur CL, Thuillier P, et al. Case report: Eosinophilic fasciitis induced by pembrolizumab with high FDG uptake on 18F-FDG-PET/CT. *Frontiers in Medicine*. 2022;9. doi:10.3389/fmed.2022.1078560
3. Bickel A, Koneth I, Enzler-Tschudy A, Neuweiler J, Flatz L, Früh M. Pembrolizumab-associated minimal change disease in a patient with malignant pleural mesothelioma. *BMC Cancer*. 2016;16(1). doi:10.1186/s12885-016-2718-y
4. Bourcier L, St-Hilaire È, LeBlanc M, Picard L. Complete reversibility of pembrolizumab-induced eosinophilic fasciitis without corticosteroids: A case report. *SAGE Open Medical Case Reports*. 2021;9. doi:10.1177/2050313x211025111
5. Gauci ML, Baroudjian B, Laly P, et al. Remitting seronegative symmetrical synovitis with pitting edema (RS3PE) syndrome induced by nivolumab. *Seminars in Arthritis and Rheumatism*. 2017;47(2):281-287. doi:10.1016/j.semarthrit.2017.03.003
6. Kobak S. Pembrolizumab-Induced Seronegative Arthritis and Fasciitis in a Patient with Lung Adenocarcinoma. *Current Drug Safety*. 2019;14(3):225-229. doi:10.2174/1574886314666190528121039
7. Moriyama S, Fukata M, Tatsumoto R, Kono M. Refractory constrictive pericarditis caused by an immune checkpoint inhibitor properly managed with infliximab: a case report. *European Heart Journal - Case Reports*. 2021;5(1). doi:10.1093/ehjcr/ytab002
8. Olamiju B, Odell I, Panse G, Eder JP, Leventhal JS. Skin puckering and edema during durvalumab therapy. *JAAD Case Reports*. 2020;7:110-112. doi:10.1016/j.jdcr.2020.11.019
9. Pabón-Cartagena G, López A, Watts E, Alonso N. Eosinophilic fasciitis in association with nivolumab: The importance of eosinophilia. *JAAD Case Reports*. 2020;6(12):1303-1306. doi:10.1016/j.jdcr.2020.04.017
10. Redman JM, Rhea LP, Cordes L, et al. A case of Anti–PD-L1-associated remitting seronegative symmetric synovitis with pitting edema. *Clinical Genitourinary Cancer*. 2019;17(3):e549-e552. doi:10.1016/j.clgc.2019.02.005
11. Tajmir-Riahi A, Bergmann T, Schmid M, Agaimy A, Schuler G, Heinzerling L. Life-threatening autoimmune cardiomyopathy reproducibly induced in a patient by checkpoint inhibitor therapy. *Journal of Immunotherapy*. 2017;41(1):35-38. doi:10.1097/cji.0000000000000190
12. Velev M, Baroudjian B, Pruvost R, et al. Immune-related generalised oedema – A new category of adverse events with immune checkpoint inhibitors. *European Journal of Cancer*. 2022;179:28-47. doi:10.1016/j.ejca.2022.11.001
13. Xu F, Xiao C, Sun W, et al. A lung adenocarcinoma patient with ROS1 fusion and NBN germline mutation achieves long progression-free survival from sintilimab combined with niraparib after failure of ROS1 inhibitors: a case report. *Annals of Translational Medicine*. 2022;10(16):912. doi:10.21037/atm-22-3582
14. Zierold S, Akcetin LS, Gresser E, et al. Checkpoint-inhibitor induced Polyserositis with Edema. *Cancer Immunology Immunotherapy*. 2022;71(12):3087-3092. doi:10.1007/s00262-022-03211-7
15. Bui ATN, Nelson CA, Lian CG, Canales AL, LeBoeuf NR. Eosinophilic fasciitis induced by nivolumab therapy managed without treatment interruption or systemic immunosuppression. *JAAD Case Reports*. 2020;6(8):693-696. doi:10.1016/j.jdcr.2020.04.010
16. Chan KK, Magro C, Shoushtari A, et al. Eosinophilic fasciitis following checkpoint inhibitor therapy: Four cases and a review of literature. *The Oncologist*. 2019;25(2):140-149. doi:10.1634/theoncologist.2019-0508
17. Herrera AF, Goy A, Mehta A, et al. Safety and activity of ibrutinib in combination with durvalumab in patients with relapsed or refractory follicular lymphoma or diffuse large B‐cell lymphoma. *American Journal of Hematology*. 2019;95(1):18-27. doi:10.1002/ajh.25659
18. Kim ST, Murphy WA, Aparicio A, Subudhi SK. RS3PE following treatment with combination of hormonal therapies plus ipilimumab in a patient with metastatic prostate cancer. *Journal of Immunotherapy and Precision Oncology*. 2020;3(3):128-132. doi:10.36401/jipo-20-2
19. Murakami S, Nagano T, Nakata K, et al. Tenosynovitis induced by an immune checkpoint inhibitor: A case report and literature review. *Internal Medicine*. 2019;58(19):2839-2843. doi:10.2169/internalmedicine.2556-19
